# *Helicobacter pylori* outer membrane vesicles induce astrocyte reactivity through nuclear factor-κappa B activation and cause neuronal damage in vivo in a murine model

**DOI:** 10.1186/s12974-023-02728-7

**Published:** 2023-03-09

**Authors:** Esteban Palacios, Lorena Lobos-González, Simón Guerrero, Marcelo J. Kogan, Baohai Shao, Jay W. Heinecke, Andrew F. G. Quest, Lisette Leyton, Manuel Valenzuela-Valderrama

**Affiliations:** 1 Laboratorio de Microbiología Celular, Instituto de Investigación y Postgrado, Facultad de Ciencias de La Salud, Universidad Central de Chile, 8330546 Santiago, Chile; 2 Laboratory of Cellular Communication, Center for Studies On Exercise Metabolism and Cancer (CEMC), Institute of Biomedical Sciences (ICBM), Facultad de Medicina, Universidad de Chile, 8380453 Santiago, Chile; 3 Advanced Center for Chronic Diseases (ACCDiS), Facultad de Ciencias Químicas y Farmacéuticas, Universidad de Chile, 8380494 Santiago, Chile; 4 Departamento de Química Farmacológica y Toxicológica, Facultad de Ciencias Químicas y Farmacéuticas, Universidad de Chile, 8380494 Santiago, Chile; 5 Division of Metabolism, Endocrinology and Nutrition, University of Washington, Seattle, WA 98195-8055 USA; 6 Centro de Medicina Regenerativa, Facultad de Medicina, Universidad del Desarrollo-Clínica Alemana, 7590943 Santiago, Chile; 7 Facultad de Medicina, Universidad de Atacama, 153601 Copiapó, Chile

**Keywords:** OMVs, *Helicobacter pylori*, Astrogliosis, Inflammation, Brain damage, NF-κB

## Abstract

**Background:**

*Helicobacter pylori (Hp)* infects the stomach of 50% of the world’s population. Importantly, chronic infection by this bacterium correlates with the appearance of several extra-gastric pathologies, including neurodegenerative diseases. In such conditions, brain astrocytes become reactive and neurotoxic. However, it is still unclear whether this highly prevalent bacterium or the nanosized outer membrane vesicles (OMVs) they produce, can reach the brain, thus affecting neurons/astrocytes. Here, we evaluated the effects of *Hp* OMVs on astrocytes and neurons in vivo and in vitro.

**Methods:**

Purified OMVs were characterized by mass spectrometry (MS/MS). Labeled OMVs were administered orally or injected into the mouse tail vein to study OMV-brain distribution. By immunofluorescence of tissue samples, we evaluated: GFAP (astrocytes), βIII tubulin (neurons), and urease (OMVs). The in vitro effect of OMVs in astrocytes was assessed by monitoring NF-κB activation, expression of reactivity markers, cytokines in astrocyte-conditioned medium (ACM), and neuronal cell viability.

**Results:**

Urease and GroEL were prominent proteins in OMVs. Urease (OMVs) was present in the mouse brain and its detection coincided with astrocyte reactivity and neuronal damage. In vitro, OMVs induced astrocyte reactivity by increasing the intermediate filament proteins GFAP and vimentin, the plasma membrane α_V_β_3_ integrin, and the hemichannel connexin 43. OMVs also produced neurotoxic factors and promoted the release of IFNγ in a manner dependent on the activation of the transcription factor NF-κB. Surface antigens on reactive astrocytes, as well as secreted factors in response to OMVs, were shown to inhibit neurite outgrowth and damage neurons.

**Conclusions:**

OMVs administered orally or injected into the mouse bloodstream reach the brain, altering astrocyte function and promoting neuronal damage in vivo. The effects of OMVs on astrocytes were confirmed in vitro and shown to be NF-κB-dependent. These findings suggest that *Hp* could trigger systemic effects by releasing nanosized vesicles that cross epithelial barriers and access the CNS, thus altering brain cells.

**Supplementary Information:**

The online version contains supplementary material available at 10.1186/s12974-023-02728-7.

## Background

*Helicobacter pylori* (*Hp*) colonizes the gastric epithelium of ~ 50% of the world’s population and is more prevalent in developing countries [1, 2]. This pathogen is acquired during childhood and persists in the stomach for life without antibiotic therapy [3, 4]. Chronic infection is mostly asymptomatic, but occasionally, its presence favors the development of severe gastric and duodenal pathologies, even gastric cancer [5–7]. Moreover, recent evidence points towards the existence of a direct relationship between *Hp* infection and several extra-gastric pathologies, including neurodegenerative disorders [7–9].

Case–control studies have revealed a relationship between *Hp* and the severity of Alzheimer’s and Parkinson’s disease [10, 11]; however, this bacterium is characterized by a poor invasiveness, limited only to gastric cells [12, 13]. Several hypotheses have suggested how *Hp* may reach the brain, promote inflammation, and predispose to neurodegenerative diseases [8], but experimental data are only just emerging [14]. Mice infected with *Helicobacter suis, Helicobacter pylori,* or *Helicobacter felis* show signs of neuroinflammation after 1, 5, or 18 months post-infection, respectively [14–16]. On the other hand, the *Hp*-derived peptide RpL1 aa 2–20 [Hp (2–20)] modulates the expression of hallmark genes of Alzheimer’s disease in MKN28 gastric cells, including many genes involved in inflammation [17]. Therefore, this pathogen could potentially affect not only the gastrointestinal tract, but also the central nervous system (CNS). However, the mechanism through which *Hp* causes CNS damage is unknown.

Secreted outer membrane vesicles (OMVs) have emerged as a relevant factor in bacterial pathogenesis. Like other Gram-negative bacteria, *Hp* secretes OMVs as part of its normal growth, both in vitro and in vivo [18–20] and as proposed earlier, these vesicles may play a role in the progression of *Hp*-associated gastric pathologies [12]. Therefore, OMVs could function as factors that amplify the virulence of this poorly invasive bacteria [12, 21]. OMVs are spherical, bi-layered membrane-derived nanosized vesicles with a diameter of 20–450 nm [22]. OMVs are rich in outer membrane proteins, lipopolysaccharides (LPS), glycerophospholipids, and periplasmic components, and also contain various cargos, including cytosolic proteins, inner membrane proteins, and nucleic acids (DNA and RNA) [23].

*Hp* infection disrupts the gastric epithelial barrier by altering the tight junction proteins occludin, claudin-4, and claudin-5 in the absence of host inflammatory cells [24]. In addition, when mice were inoculated orally with fluorescently labeled *Hp* OMVs or bacteria, only the OMVs were retained in the stomach for an extended period of time [25]. Related studies, in which labeled *Porphyromonas gingivalis* OMVs were orally administered to mice, have also shown decreased tight junction-related gene expression in the brain, activation of glial cells, and impaired mouse memory and learning ability [26]. A different study using mice injected with OMVs isolated from feces of Alzheimer’s disease patients or from periodontopathogen *Aggregatibacter actinomycetemcomitans* cultures revealed that OMVs can cross and damage the blood–brain barrier (BBB), promote neuroinflammation, and cause cognitive impairment [27, 28]. However, experimental evidence describing the effects of *Hp* OMVs in brain cells is still missing. Of note, a recent review included some preliminary results, showing that *Hp*-infected mice exhibited antigens in the stomach, but not in the brain. This study also found *Hp* DNA in the blood of infected mice 5 months after infection. Additionally, using fluorescently labeled *Hp* OMVs injected into the mouse tail vein, the authors demonstrated that the fluorescence was transferred to the brain, and activated the microglia [14]; however, the impact of these OMVs on astrocytes, the reactive phenotype, function, and the subsequent effects in neurons, was not addressed.

Astrocytes are the most abundant glial cells in the CNS that establish close contact with neurons, blood vessels, other glial cells, and other astrocytes [29]. Astrocytes modulate and maintain the BBB and brain homeostasis, and support neuronal function by providing a cellular link between the neuronal network and blood vessels [30]. When exposed to pro-inflammatory molecules, such as the tumor necrosis factor (TNF; aliases are TNFSF2, TNFA, and TNFα), injury, or ischemic stroke, astrocytes acquire a reactive phenotype, suffering dramatic morphological changes and overexpressing specific proteins [31, 32]. Reactive astrocytes are found during astrogliosis, a process that exerts both beneficial and harmful effects on brain homeostasis [33]. In pathological astrogliosis, produced by neurotoxic A1 astrocytes [32, 34, 35], specific reactivity markers are expressed, including connexin 43, vimentin, and glial fibrillar acidic protein (GFAP) [36, 37]. Another crucial event in astrogliosis and neurotoxicity is the activation of nuclear factor *kappa* B (NF-κB), since inhibition of NF-κB ameliorates astrogliosis and neuronal loss in several models [38–40].

Persistent *Hp* infection is associated with moderate and severe chronic inflammation [41]. OMVs induced the production of cytokines and chemokines, recruiting immune cells to the infection site [21]. Since *Hp* infection and OMVs are related to inflammation and astrocytes become reactive under inflammatory conditions, we hypothesized that nanosized *Hp* OMVs could reach the brain, induce astrogliosis, and provoke neuronal damage. To our knowledge, no evidence has shown yet a link between *Hp* OMVs and astrocyte reactivity, nor that NF-κB is involved in OMV-induced astrogliosis. Therefore, this study sought to evaluate whether *Hp* OMVs could access the mouse brain when administered orally or injected systemically, and test the *Hp* OMV effects on astrocytes and neurons in vivo. We additionally studied the molecular mechanisms through which *Hp* OMVs induce astrocyte reactivity in vitro. In a murine in vivo model, our findings show that *Hp* OMVs can reach the brain, which shows astrocyte reactivity and damaged neurons. We also demonstrate that *Hp* OMVs induce astrocyte reactivity by increasing the expression of various reactivity markers, astrocyte migration, and IFNγ secretion. Such changes lead to altered neuronal function in vitro*.* Moreover, we show that some of these OMV effects on astrocytes are NF-κB-dependent.

## Methods and experimental models

### Mice

Male BALB/c mice (6–8 weeks) were kept under controlled temperature (21 ± 2 °C) and light (12 h/12 h light/dark cycle). Mice were fed with a standard chow diet for rodents and drank water ad libitum. OMVs were administered by either tail vein injection or oral gavage. Mice were injected via the tail vein with different doses of *Hp* OMVs (5, 10, 20 or 100 µg), diluted in physiological serum (250 µl). For the analysis of OMVs biodistribution, vesicles were labeled with DiR (1,1′-dioctadecyl-3,3,3′,3′-tetramethylindotricarbocyanine iodide, Thermo Fisher Scientific, Waltham, MA, USA). After 24 h, mice were euthanized, and their organs were dissected. For oral gavage, mice were fasted for 12 h before inoculation. Then, 100 µg of DiR-OMVs were diluted in 250 µl of PBS (total volume) and administrated using a feeding tube (20GA, 38 mm, Thermo Fisher Scientific). After 24, 48, and 72 h, mice were euthanized. The accumulated fluorescence was immediately evaluated in the in vivo FX Pro system (Bruker). For analysis of brain damage in response to OMVs, mice were injected with non-labeled OMVs and euthanized after 72 h. For posterior analysis, brain tissues were fixed in 4% paraformaldehyde for 24 h and then embedded in sucrose (10 and 30%) for an additional 24 h period. Subsequently, brains embedded in a small amount of optimal cutting temperature (OCT) compound were cut into coronal tissue sections (20 µm) with a cryomicrotome (Leica CM1860 UV), mounted on positively charged microscope slides (Citoglass^®^) and kept at – 80 °C until use. The integrity of tissue sections was evaluated by haematoxylin/eosin staining (Thermo Fisher Scientific) of consecutive slices, as previously described [42]. All animal protocols were approved by the Ethics Committee of Universidad de Chile (CBA-FMED# 1123) and Universidad Central de Chile (Project #28/2021).

### Bacteria

The *Hp* 60190 strain (ATCC 49503) was grown in Brucella broth, supplemented with 0.3% β-cyclodextrin (Sigma-Aldrich), *Hp* selective supplement Dent (Oxoid) and Isovitalex (Oxoid), under microaerophilic conditions at 37 °C for 72 h. Bacteria were first grown to an optical density of 0.6–1.0 at 600 nm (OD_600_) and subsequently diluted to a starting OD_600_ of 0.05, as previously described [43, 44].

### Cells

The immortalized catecholaminergic neuronal cells CAD were cultured in DMEM-F12 (Gibco) media, supplemented with 8% fetal bovine serum (FBS) and antibiotics (100 UI/ml ampicillin and 100 µg/ml streptomycin) in a humidified atmosphere and 5% CO_2_ at 37 °C.

DITNC1 rat astrocytes were cultured in RPMI 1640 (Gibco) media, supplemented with 5% FBS, 0.1 mM 2-mercaptoethanol, and antibiotics at 37 °C in a humidified incubator and 5% CO_2_. Cells were subcultured between passages 9–14.

Primary astrocytes were obtained from the cortex tissue of Wistar neonatal rats (P0–P1). Tissues were treated with trypsin and mechanically disrupted to obtain the cells, which were cultured in DMEM-F12 media, supplemented with 10% FBS and antibiotics. Once confluent, cells other than astrocytes were removed by shaking in an orbital shaker at 180 rpm overnight [36].

### Purification of Hp OMVs

*Hp* OMVs were purified following previously described protocols with minor modifications [27, 45]. Briefly, 200 ml *of Hp* liquid cultures were centrifuged at 2200 × g for 10 min at 4 °C. Then, the supernatant was recovered, passed through 0.2 µm cellulose acetate filters, and finally concentrated using the Amicon 100 kDa columns (Merck), following the instructions provided by the manufacturer. Next, OMVs were precipitated by mixing 1 volume of concentrated media (approximately, 1.5 ml) with 0.5 volume of the Exo-spin precipitation buffer #EX-01 (Cell Guidance System LLC, Helix Center, USA) for 1 h, at 4 °C, and then centrifuged at 16000 × g and 4 °C for 1 h. The vesicle pellets were resuspended in 100 µl of phosphate buffered saline (PBS) pH 7.2 and further purified using an exclusion chromatography column (Invitrogen Exosome Spin Column MW3000). The protein concentration of the OMVs preparations was quantified using the BCA protein assay kit (Pierce Thermo Scientific, Rockford, IL, USA).

### Characterization of the OMVs

The size distribution and concentration of the OMV preparations were determined by nanoparticle tracking analysis (NTA), using NanoSight^®^ NS300 (Malvern Panalytical). Samples were diluted at 1:100 in PBS to reach the desired concentration of 100–1000 particles per captured image. Additionally, OMVs were characterized by transmission electron microscopy (TEM). Samples were deposited on FCF300-Cu grids (Sigma-Aldrich), fixed with 1% glutaraldehyde for 5 min, and dried with absorbent paper. Images were captured in a Philips Tecnai 12-BioTwin transmission electron microscope (available at Pontificia Universidad Católica de Chile). Protein extracts from OMVs were separated by 10% SDS-PAGE and gels were stained with Coomassie Blue (R-250). Gel images were acquired using the ImageQuant LAS 500 system (GE Healthcare Biosciences). The proteomic signature of OMVs was obtained by shotgun LC–ESI–MS/MS in the positive ion mode, with a precise ultrahigh resolution Orbitrap Fusion Lumos Tribrid™ mass spectrometer (Thermo Fisher Scientific) coupled to a nanoACQUITY UPLC System (Waters Corporation), as previously described [46]. Proteins extracted from vesicles were trypsin digested after reduction with dithiothreitol and alkylation with iodoacetamide. Digested peptides were separated using a C-18 analytical column (0.1 × 200 mm) in-house packed with Magic C-18 reverse-phase resin (5 µm; 100 Å; Michrom Bioresources). Linear gradients of 0.1% formic acid in water (solvent A) and 0.1% formic acid in acetonitrile (solvent B) were used for the separation. The MS/MS spectra were compared with the UniProtKB database of *Hp* proteins (UP000000429.fasta), using the Comet MS/MS search engine (version 2018.01 rev.2) with fixed Cys alkylation and variable Met oxidations. Results were validated with the PeptideProphet and ProteinProphet Softwares, using an adjusted probability of 0.90 for peptides and 0.95 for proteins. For the identification of a protein, at least two peptides unique to the protein of interest had to be detected. Total peptide counts were used to compare protein abundance.

### Indirect immunofluorescence

For immunofluorescence analysis of astrocytes, cells were seeded at a density of 4 × 10^3^/cm^2^ in 24-well plates on circular cover glasses pretreated with a 0.1% (w/v) poly-lysine solution in water (Sigma) for 5 min. After 24 h of culture, the cells were subjected to the experimental procedures. The medium was then eliminated, cells were fixed with 4% paraformaldehyde, washed three times with PBS, permeabilized with 0.1% Triton X-100 in PBS (15 min; room temperature), and finally blocked with 5% BSA. Samples were incubated with the corresponding primary antibodies for 1 h at room temperature. Next, samples were washed three times with PBS. Then, samples were incubated with the secondary antibody and DAPI (0.025 µg/ml) for 1 h at room temperature. Finally, the samples were washed three times with PBS for 5 min and mounted on slides with 8 µl of Fluoromount^TM^ (Sigma-Aldrich). For immunofluorescence analysis of brain tissues, slides were thawed and rinsed with PBS to eliminate residual material. Permeabilization was performed with 1% Triton X-100 for 10 min in a humidified chamber. Subsequently, slides were incubated with blocking solution (0.5% horse serum, 2% de BSA, and 0.5% Triton X-100 in PBS) for 1 h and then incubated overnight at 4 °C with the corresponding primary antibody diluted in the same blocking solution. Slides were washed three times with 0.5% horse serum in PBS for 5 min at room temperature. Subsequently, the samples were incubated with the secondary antibody, prepared in 0.5% horse serum in PBS, for 2 h in a dark, humid chamber. The samples were then washed five times with PBS for 5 min, finally mounted with Fluoromount^TM^, and analyzed using a Nikon C2 plus Spectral confocal microscope, with a 40X objective and the ImageJ Software.

### Immunoblot analysis

Total protein extracts were obtained by sonication in a buffer containing 20 mM HEPES (pH 7.4), 0.1% SDS, 0.05% NP-40, and a cocktail of protease inhibitors (Roche). Samples were centrifuged (11000 × g, 4 °C) and supernatants were collected and stored at – 80 °C. Total proteins (50 μg per lane) were separated by 10% SDS-PAGE and transferred to nitrocellulose membranes (Amersham), as previously described [47]. Membranes were blocked with PBS containing 0.1% Tween and 5% skimmed milk for 1 h and incubated with primary antibodies diluted in the same buffer. Primary antibodies were detected with secondary antibodies conjugated with HRP and the EZ-ECL kit (Biological Industries). Images were captured with an ImageQuant LAS 500 imager (General Electric Healthcare Biosciences). Protein levels were quantified by densitometric analysis using the NIH ImageJ Software [48]. The primary and secondary antibodies that we used are listed in Additional file 1: Table S2.

### DEVDase activity

Caspase 3 activity was determined by following the release of the fluorescent dye 7-amino-4-trifluoromethylcoumarin (AFC) from the substrate Asp-Glu-Val-Asp-AFC (Enzo Life Sciences) in cell lysates. Fluorescent readouts (λexc = 375 nm, λem = 530 nm) were quantified using an Infinite M200Pro (TECAN) multiplate reader. A unit of enzymatic activity was defined as 1 mmol of substrate transformed per min per mg of protein extract, as previously described [49].

### Flow cytometry analysis

Apoptosis was evaluated by flow cytometry (BD FACSCanto) using the FITC Annexin V Apoptosis Detection Kit I (BD Pharmingen™), following the instructions provided by the manufacturer. Briefly, differentiated CAD cells (48 h) were treated with the ACM media or Etoposide 10 µM for 24 h [50], and then harvested by trypsinization for 1 min at 37 °C. Following two rounds of washes with PBS, cells were resuspended in the binding buffer and incubated with PI and Annexin V-Alexa Fluor 488 for 30 min. Data were processed using the FlowJo v10.8.0 Software [51].

### In vitro wound-healing assay

The wound-healing assays were performed as previously described [52]. Briefly, primary astrocytes were seeded at a density of 1 × 10^5^ cells per well in a 24-well plate and grown to 90% confluency. Following 18 h of culture, astrocytes were preconditioned with either OMVs (2.5 µg/ml) for 12 h, TNF (10 ng/ml) for 48 h or left untreated. Next, the wounds were made with a sterile micropipette tip (p10), and detached cells were eliminated by washing twice with PBS. The medium was then replaced with 500 µl of SFM, and after 30 min, five images were obtained per well (Time 0 h). Astrocytes were then treated with either Thy-1-Fc/Protein-A or TRAIL-R2-Fc/Protein-A complexes at a concentration of 4 μg/0.4 μg per well, and non-stimulated astrocytes were maintained in SFM as basal controls. As a positive control, astrocytes were stimulated with 3% FBS. After 24 h of stimulation, five images were obtained for each condition (Time: 24 h). Images were captured in a Motic^TM^ Trinocular AE31E microscope and analyzed with the NIH ImageJ Software, using the Area Calculator Plugin. The wound area was calculated using the equation: % wound closure = [(A_0h_ − A_24h_)/A_0h_] × 100, where A_0h_ is the wound area produced immediately after scratching (Time 0 h) and A_24h_ is the wound area at 24 h post-scratching.

### NF-κB transcriptional activity

NF-κB transcriptional activity was determined using the NF-κB (p65) Transcription Factor Assay Kit (Cayman Chemical #10007889). Primary astrocytes treated with OMVs (2.5 µg/ml, 12 h) or TNF (10 ng/ml, 48 h) were incubated for 60 min with or without the IKK (IMD 0354 [53] or BMS 345541 inhibitors [54]). Nuclear extracts were obtained from treated cells as previously described [49]. A volume of 10 µl containing normalized nuclear protein extracts was added per well, and adsorbed p65 was quantified following the manufacturer’s instructions.

### Morphological differentiation of neuronal CAD cells over fixed DITNC1 astrocytes

DITNC1 astrocytes were seeded at a density of 5 × 10^5^ cells/cm^2^ and incubated to 90% confluency in 6-well glass bottom plates. Then, the cells were treated with OMVs (2.5 μg/ml) or TNF (10 ng/ml) for 12 h or 48 h, respectively. Next, the medium was eliminated, and cells were washed twice with PBS and fixed with 4% paraformaldehyde for 15 min. The reaction was then stopped with 0.1 mM glycine. Following extensive washing with PBS, CAD cells labeled with Cell Tracker Green CMFDA (10 µM) were seeded over fixed astrocytes at a density of 1 × 10^4^ cell/cm^2^ in DMEM F12 media, supplemented with 8% FBS and antibiotics [55]. Before 24 h of culture, the media was replaced with fresh SFM containing 50 ng/ml sodium selenite (S5261, Sigma-Aldrich) to induce morphologically visible neuronal differentiation. After 24 h, neuronal processes were captured with an Epifluorescent Spinning disk microscope (Olympus). The neurite extension length was determined using the NeuronJ plug-in of the v1.8 NIH ImageJ Software, as previously described [56, 57].

### Astrocyte-conditioned medium

Astrocytes were seeded at a density of 3 × 10^5^ cells/cm^2^ in 6-well plates and grown to 90% confluency for 24 h. The cells were treated with 2.5 µg/ml of OMVs for 12 h or 10 ng/ml of TNF for 48 h, washed with PBS to eliminate free OMVs and TNF, and then incubated with SFM for 5 days. The astrocyte-conditioned media (ACM) were filtered through 0.2 µm cellulose acetate filters and stored frozen at – 80 °C, until required.

### Multiplex assay

The collected ACM was used to quantify TNF, IL-6, IL-1β, and IFNγ using the MILLIPLEX MAP RAT cytokine/chemokine magnetic bead panel RECYTMAG-65K (Merck), following the manufacturer’s instructions. Data acquisition and analysis were performed in the LUMINEX platform using the Q-View (Quansys Biosciences) Software.

### Statistical analysis

The experiments were repeated at least 3 times, independently. When comparing two groups, the confidence limits were established by comparing the percentiles using the *t*-Student distribution (two tails) or the Mann–Whitney test. Two-way ANOVA analysis was used to compare three or more experimental groups, followed by a post hoc comparison with the Bonferroni test. Data are expressed as the mean ± standard error of the mean (S.E.M.) in all cases. A probabilistic value of *p* < 0.05 was considered significant. The software used for the statistical analysis was GraphPad prism V9.

## Results

### Characterization of Hp outer membrane vesicles

**Fig. 1:**
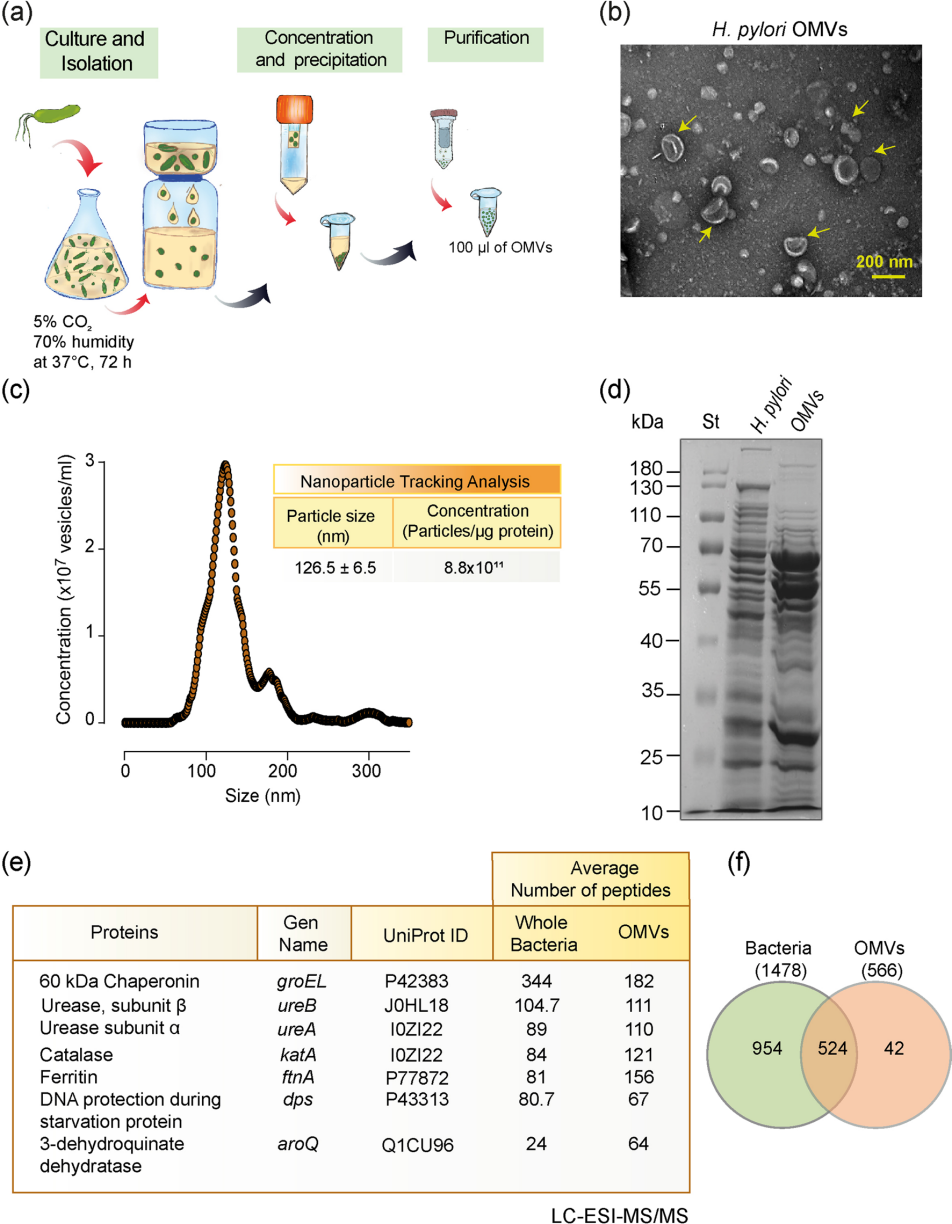
Characterization of *Hp* 60190 outer membrane vesicles. **a** General scheme of the protocol for the isolation, concentration, and purification of *Hp* OMVs. **b** *Hp* OMVs observed by transmission electron microscopy. The yellow arrow indicates *Hp* vesicles (scale bar = 200 nm). **c** Nanoparticle tracking analysis (left) shows average concentration of OMVs (vesicles/ml) of a particular size (nm). Values in the table correspond to the quantification obtained by the nanoparticle tracking analysis (mean ± S.E.M.; *n* = 3) (right). **d** Electrophoretic profile of *Hp* 60190 total protein extracts and OMVs resolved on 12% SDS-PAGE and stained with Coomassie blue. St indicates molecular marker standards (kDa, left). **e** LC–ESI–MS/MS proteome analysis of whole bacteria and purified OMVs. The table shows the proteins identified as having the most abundant unique peptides (*n* = 3). **f** Venn diagram showing the number of proteins detected in the whole bacteria and purified OMVs

We first obtained OMVs from liquid cultures of the *Hp* strain 60190 (ATCC 49503) (Fig. 1a). Structural integrity of *Hp* OMVs was confirmed by transmission electron microscopy (Fig. 1b). Nanoparticle tracking analysis (NanoSight) indicated that the OMVs had an average diameter of 126.5 ± 6.5 nm and a concentration of 8.8 × 10^11^ particles/μg of protein (Fig. 1c). After comparing the total protein profile of *Hp* OMVs with the whole extract of the parental bacterium, we found that the protein profile of OMVs was different from that of the parental bacterial extract and showed enriched protein bands (Fig. 1d). Then, we assessed the relative abundance and proteomic profile of the OMVs by liquid chromatography–mass spectrometry (LC–MS/MS). The proteins with the most abundant unique peptides were the 60-kDa chaperonin (GroEL), urease (UreA and UreB), catalase, and ferritin (Fig. 1e). We identified 1478 proteins in the whole bacteria lysates and 566 proteins in OMVs, of which 524 were common to whole bacteria and the OMVs (Fig. 1f). All proteins detected as containing unique peptides in OMV samples [42] are listed in Additional file 1: Table S1. This table includes relevant virulence factors, such as VacA, SabA, Cag5, and enzymes related to both energy and DNA metabolism, like GDP-L-fucose synthase, N-acetyl galactosamine epimerase, and site-specific DNA-methyltransferase. These results suggest that *Hp* OMVs possess an abundant cargo of virulence factors and enzymes typically found in the parental bacterium. Therefore, due to their small size and content, OMVs could play a role in long-distance signaling and some of the extra-gastric, systemic effects of *Hp* infection.

### Systemically administered Hp OMVs reach the brain in BALB/c mice

**Fig. 2:**
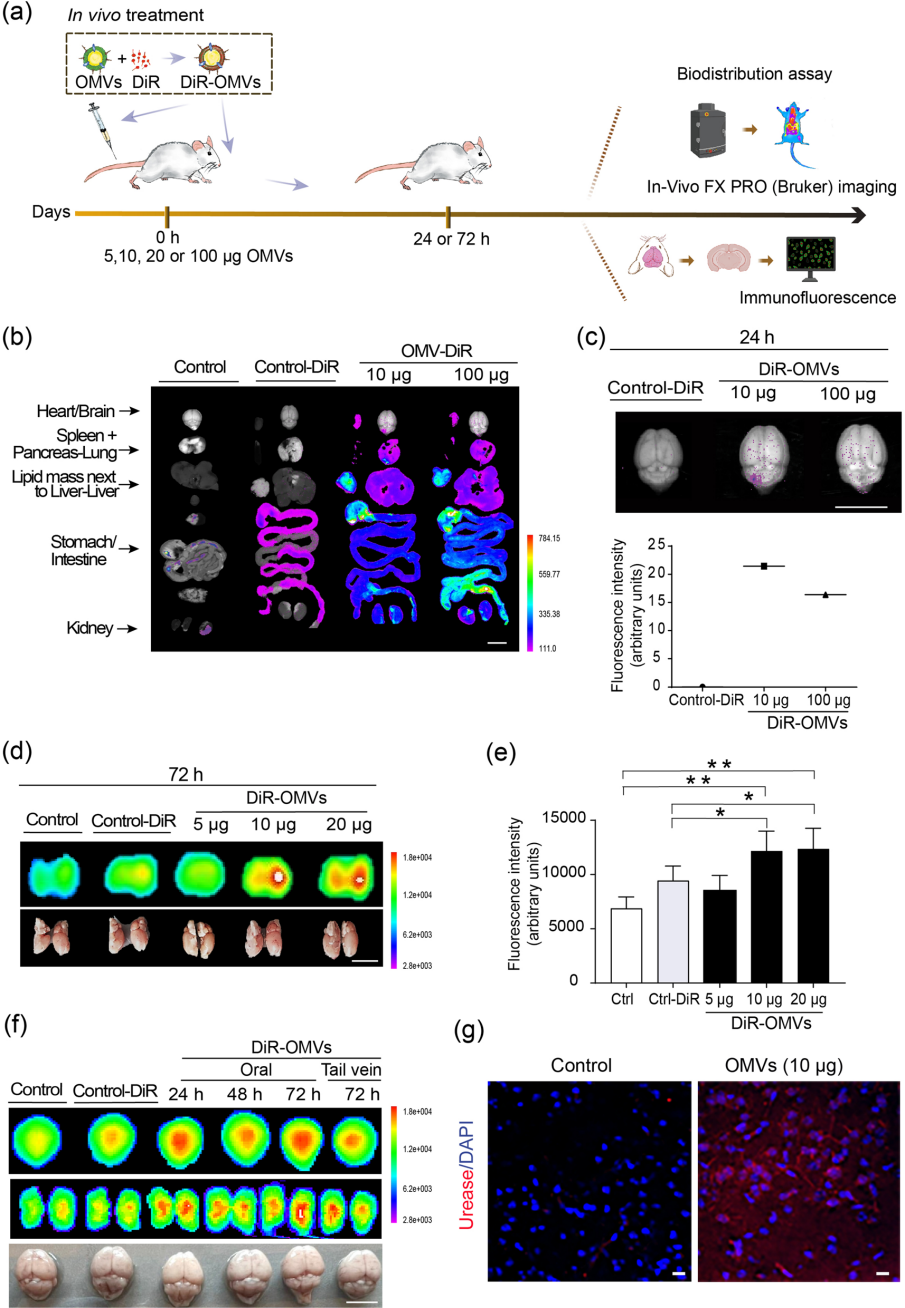
*Hp* OMVs administered systemically or orally reach the brain in BALB/c mice. **a** Scheme of the in vivo experimental design. DiR-labeled *Hp* OMVs were administered to Male BALB/c mice orally or via the tail vein. The biodistribution of labeled OMVs was evaluated using the FX PRO (Bruker) imaging system. **b** Ex vivo fluorescence images of dissected organs following different treatments. Mice injected via the tail vein with control, control-DiR, 10 or 100 µg of DiR-OMVs, were killed after 24 h for optical imaging (scale bar = 1 cm). **c** Quantification of fluorescence intensity (arbitrary units) in the mouse brain following the treatments indicated in **b**. **d** Ex vivo fluorescence images of brain hemispheres following the indicated treatments, 72 h after injection with 5, 10, or 20 µg of DiR-OMVs into the tail vein (scale bar = 1 cm). **e** Quantification of fluorescent signal intensities of the experiment shown in **d** performed with the Bruker Molecular Imaging Software. Values in the graph represent means ± S.E.M. of fluorescence intensity (arbitrary units); *n* = 3. *p < 0.05, **p < 0.01. **f** Ex vivo fluorescence images of whole brains and their corresponding hemispheres from mice 1 to 3 days after oral gavage administration of 100 µg of DiR-OMVs (24 h, 48 h, and 72 h) (scale bar = 1 cm). For comparison, a mouse was injected with 10 µg of OMVs via the tail vein. ** g** Confocal immunofluorescence of coronal brain tissue sections of a mouse injected with 10 µg of *Hp* OMVs stained with an anti-UreB subunit antibody (red). DAPI in blue shows nuclear staining (scale bar = 20 µm)

To evaluate whether *Hp* OMVs could reach the brain, we injected 10 and 100 µg (protein content; 8.8 × 10^12^–8.8 × 10^13^ particles) of DiR-labeled OMVs into the mouse tail vein and followed their in vivo biodistribution (Fig. 2a). In mice treated with both 10 and 100 ug of OMVs for 24 h, OMVs were detected in the stomach, intestine, heart, lungs, and brain (Fig. 2b). As expected for nanoparticles [58], the OMVs were also found in the liver and spleen (Fig. 2b). The signal in the mouse brain injected with 10 µg of OMVs was higher than that injected with 100 µg (Fig. 2c). To confirm this result, we used three different doses of DiR-labeled OMVs (5, 10, and 20 µg) (Fig. 2d). These labeled OMVs were detected in sagittal sections of the brain (yellow–red area), when mice were injected with 10 and 20, but not 5 µg of OMVs, after 72 h (Fig. 2d and e). Fluorescence intensity levels with 5 µg of OMVs were similar to those of controls (Fig. 2d and e). This injection approach was thought to have robust results and avoided the interaction of the OMVs with components of the gastric lumen, such as proteolytic enzymes, hydrochloric acid, or undigested food. However, this does not represent what would occur in the natural course of infection, where the bacterium secretes OMVs in close contact with gastric epithelial cells. With this in mind, we administered a higher oral dose of DiR-OMVs (100 μg) to fasted mice. Notably, fluorescence appeared to accumulate in the mouse brain following 24–72 h of administration (Fig. 2f).

Mass spectrometry showed that urease was one of the most abundant proteins in the OMVs (Fig. 1e). Immunofluorescence of coronal tissue sections with the anti-UreB antibody revealed a positive signal only for samples obtained from OMV-injected mice (Fig. 2g). These results confirm that Hp OMVs can cross both the gastric epithelium and BBB, accumulating in the mouse brain.

### Hp OMVs induce astrocyte reactivity and neuronal damage in vivo

**Fig. 3:**
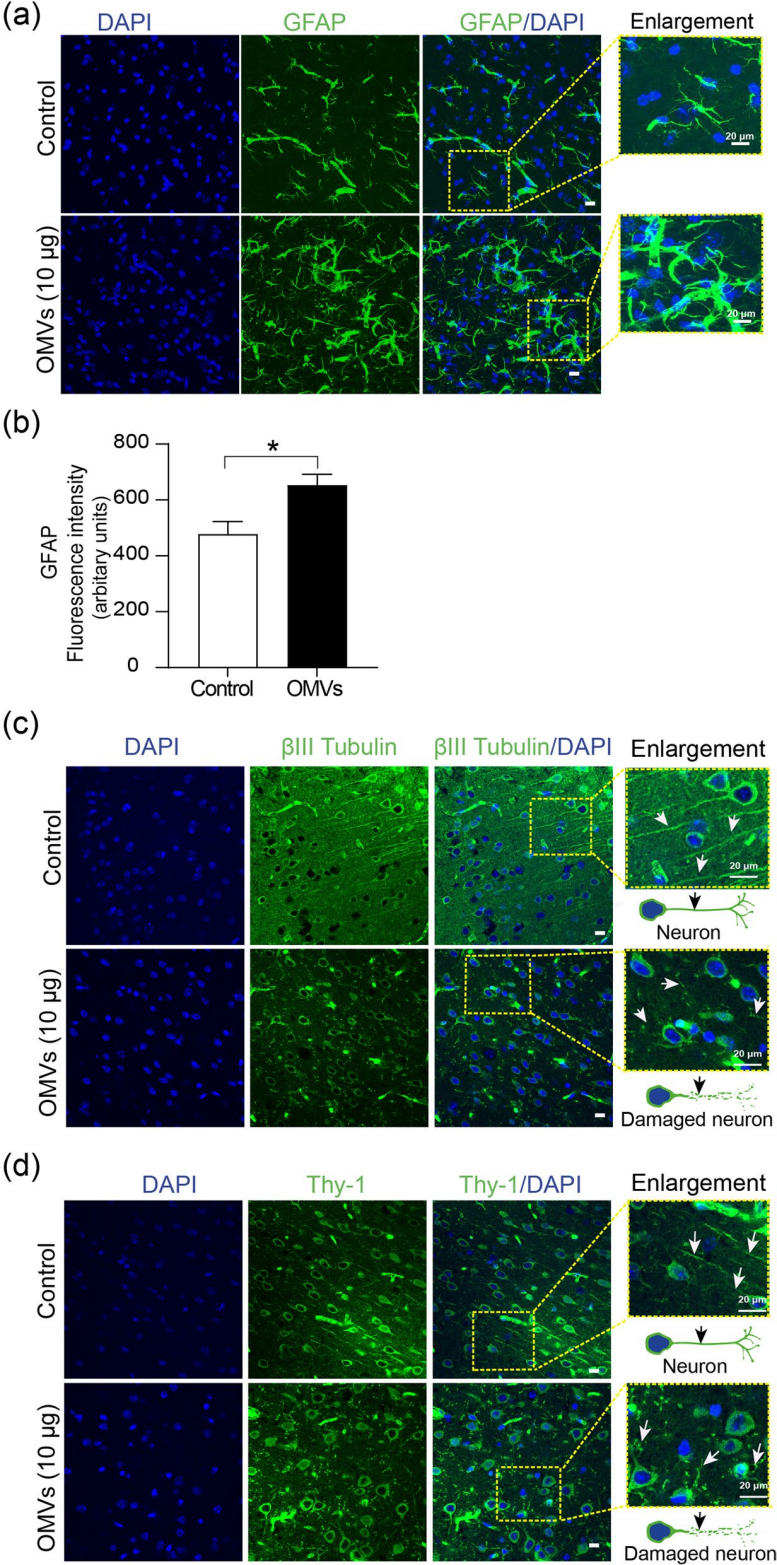
*Hp* OMVs induce astrocyte reactivity in vivo. **a** Confocal immunofluorescence of coronal brain sections from control and OMV-injected mice (10 µg) stained with DAPI (blue), and GFAP (green). The right panel shows merged GFAP and DAPI images, and the yellow dashed square shows the digitally enlarged area (scale bar = 20 µm). **b** GFAP fluorescence was quantified using mean green fluorescence intensity values measured with ImageJ (7 different fields) (scale bar = 20 µm). Values in the graph represent means ± S.E.M.; *n* = 3. *p < 0.05. **c** Confocal image, as described in **a** but stained with an anti-βIII tubulin antibody. **d** Confocal image as described in **a** but stained with an anti-Thy-1 antibody. White arrowheads in the enlarged pictures in **c**, **d** show extended or fragmented neuronal processes. Graphic representations of a neuron with an axon (black arrowhead) and dendrites (Control), and a neuron with fragmented staining of its processes due to cell damage (+ OMVs) are included

The effect of OMVs in the brain was analyzed by immunofluorescence and confocal imaging to visualize markers of astrogliosis and neuronal integrity. DAPI staining indicated a similar number of cells/nuclei in the brain tissue sections (Fig. 3a). The coronal samples obtained from mice treated with 10 µg of *Hp* OMVs for 72 h showed stellate-shaped astrocytes of larger cell size and an increase in GFAP labeling and fluorescence intensity than in controls (Fig. 3a and b), indicative of astrocyte reactivity. Similar tissue sections were stained with anti-βIII tubulin (Fig. 3c) or anti-Thy-1 antibodies (Fig. 3d). βIII tubulin is a microtubule protein of the tubulin family expressed almost exclusively in neurons and the testis [59, 60], while Thy-1 is a cell surface protein found in the axons and dendrites of mature neurons in the brain [61, 62]. Results revealed fibers and staining of neuronal soma in the cortical region of controls, whereas in OMV-treated mice, cell bodies showed less staining, and the fibers were missing. The intermediate zone between the cell bodies was filled with fragmented fibers, which may represent remnants of continuous processes (Fig. 3c and d). DAPI staining indicated a similar number of cells in the samples (Fig. 3c and d). Since the DiR-labeled OMVs reached the mouse brain, these vesicles could be responsible for the increased astrocyte reactivity shown by GFAP staining. Additionally, both OMVs and reactive astrocytes could be related to the observed neuronal damage. These results in an in vivo mouse model suggest that *Hp* OMVs reach the brain, induce astrocyte reactivity, and damage neurons.

### Hp OMVs induce a reactive phenotype in astrocytes in vitro

**Fig. 4:**
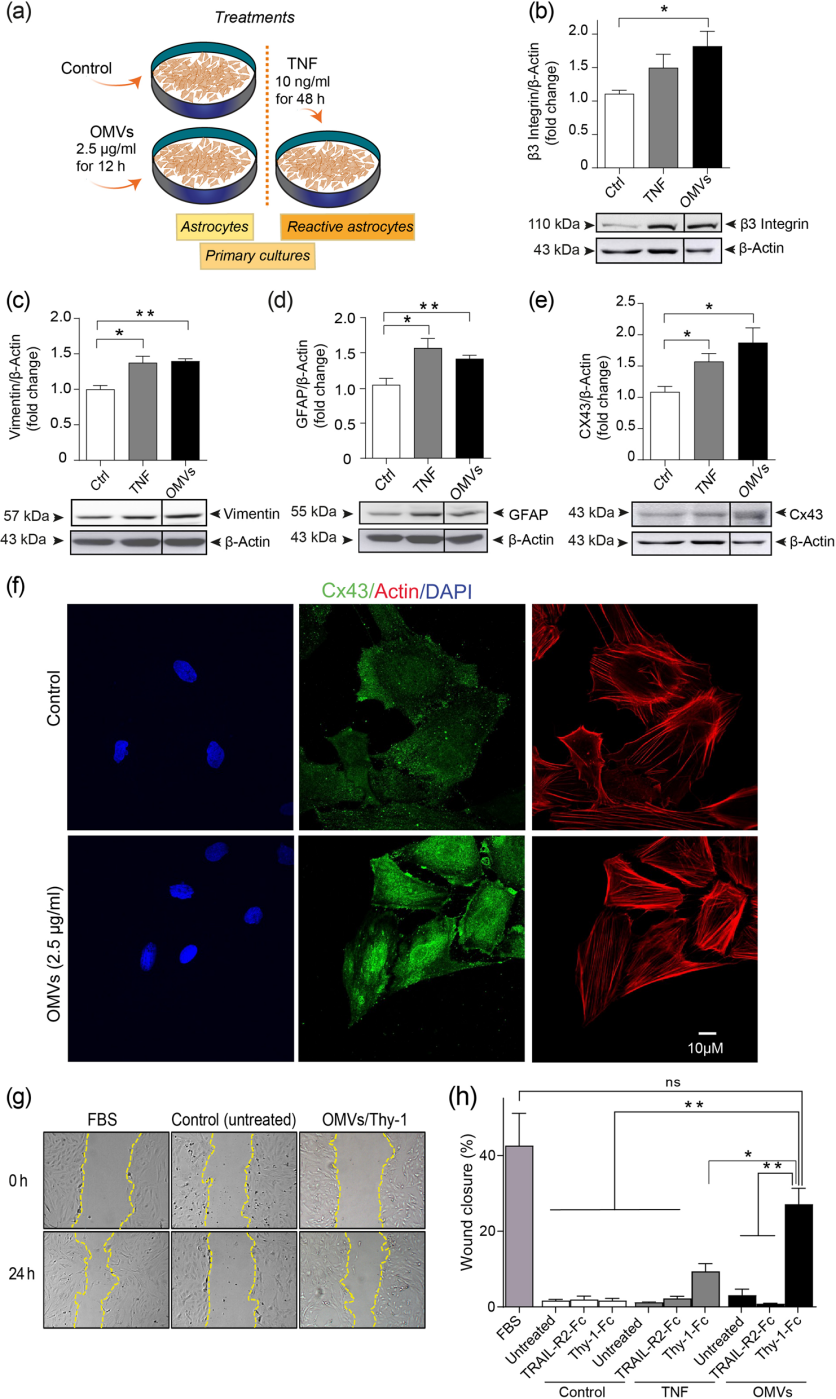
*Hp* OMVs induce a reactive astrocyte phenotype in vitro*.* **a** Scheme of various treatments. Primary astrocyte cultures were left untreated (control) or treated with 2.5 µg/ml of OMVs for 12 h or with TNF (10 ng/ml for 48 h, positive control).** b–e** The data correspond to the cell lysates from untreated astrocytes (Ctrl), treated with TNF, or with OMVs. Immunoblot analysis of β_3_ integrin (**b**), vimentin (**c**), GFAP (**d**), and connexin 43 (**e**), where β-actin is the loading control. Values in the graphs are means ± S.E.M. of the ratio between the densitometric value of the first antibody signal and that of the respective β-actin (*n* = 3). *p < 0.05, **p < 0.01. *All bands shown in each panel are from the same blot. The vertical line between bands indicates a picture cut*. **f** Confocal microscopy microphotographs of the subcellular localization of connexin 43 (Cx43, middle panel) in untreated astrocytes (control) or treated with *Hp* OMVs, which are stained for nuclei (blue), Cx43 (green), and actin cytoskeleton (red) (scale bar = 10 µm). **g** Astrocyte migration in the wound closure assay at 0 and 24 h post-scratch. Cells were pretreated or not with OMVs (2.5 µg/ml for 12 h), and then stimulated with Thy-1-Fc/protein-A (4 µg/ 0.4 µg per well) for 24 h, and cells treated with 3% FBS are the positive controls. Dashed yellow lines indicate the wound border. **h** Values in the graphs are the means ± S.E.M. of the % of wound closure in Control, TNF- or OMV-treated astrocytes stimulated or not with SFM (control), TRAIL-R2-Fc (negative control), or Thy-1-Fc. The positive control was 3% FBS (*n* = 3). ns, non-significant, *p < 0.05, and **p < 0.01

Astrocytes detect certain signals from infectious pathogens (antigens or nucleic acids), and trigger astrogliosis, which is the defensive reaction of astrocytes to acute stress [63]. To study the effects of OMVs on astrocytes and their molecular mechanisms, we used two in vitro models: the DITNC1 (ATCC) cell line and primary astrocytes from neonatal rats. As a positive control of astrocyte reactivity, DITNC1 cells were treated with 10 ng/ml TNF for 48 h to induce reactivity [36]. Time- and concentration-dependent effects of OMVs were assessed by immunoblot analysis. Connexin 43, α_V_β_3_ integrin, GFAP and vimentin levels were evaluated in DITNC1 cells treated with *Hp* OMVs (1.25–20 µg/ml) for 24 h and compared to those obtained for negative (basal levels) and positive controls. We found that 2.5 µg/ml of OMVs significantly increased (compared to basal levels) connexin 43, GFAP and vimentin levels (Additional file 1: Figure S1A, C, and D, respectively). In contrast, α_V_β_3_ integrin levels were unaltered (Additional file 1: Figure S1B). Cells were then treated with 2.5 µg/ml of *Hp* OMVs for 3, 6, 12, 24, 48, and 72 h. Treatment with OMVs increased the protein levels of connexin 43 at 3, 6, and 12 h, α_V_β_3_ integrin at 12 h, GFAP at 6–48 h, and vimentin at 3–48 h (Additional file 1: Figure S2A, B, C, and D, respectively), compared to the negative controls. Therefore, treatment with 2.5 µg/ml of *Hp* OMVs for 12 h increased expression levels of the four proteins associated with the reactive phenotype in DITNC1 astrocytes. Primary astrocytes from neonatal rats were isolated and treatment with 2.5 µg/ml OMVs for 12 h did not affect cell viability (Additional file 1: Figure S3). We then evaluated reactivity markers in primary astrocytes using the conditions mentioned above. Here, OMVs also increased connexin 43, α_V_β_3_ integrin, GFAP, and vimentin protein levels (Fig. 4a–e). These results show that treatment with 2.5 µg/ml of OMVs for 12 h induces characteristic changes in astrocytes, revealing the reactive phenotype found during astrogliosis. We corroborated these results by testing the subcellular localization of connexin 43 by indirect immunofluorescence analysis (Fig. 4f). The cytoplasmic (control) and membrane localization (+ OMVs) of connexin 43 are shown in the middle panels (green staining, Fig. 4f). Connexin 43 changed its localization from the cytoplasm to the membrane when treated with OMVs, as reported for TNF-treated astrocytes [36].

Beyond α_V_β_3_ integrin and connexin 43, reactive astrocytes express increased levels of syndecan-4, the purinergic receptor P2X7 (P2X7R), and the hemichannel pannexin 1 [36]. All these molecules are involved in migration of astrocytes when stimulated with the neuronal glycoprotein Thy-1, which engages the astrocytic proteins syndecan-4 and α_V_β_3_ integrin [36, 64, 65]. Importantly, Thy-1 promotes migration of astrocytes only when they become reactive with TNF pretreatment [36, 64, 65]. Therefore, to functionally evaluate OMV-induced astrocyte reactivity, we studied Thy-1-induced cell migration of primary astrocytes treated with OMVs or TNF. The latter was a positive control. Non-treated astrocytes (controls) or astrocytes pretreated with OMVs or TNF were stimulated with the neuronal fusion protein Thy-1-Fc or its negative control (the fusion protein TRAIL-R2-Fc, which contains the same Fc portion as Thy-1-Fc, but does not affect astrocytes) [66–68]. Cells treated with 3% FBS were positive controls for astrocyte migration. We found that primary astrocytes pretreated with OMVs in the presence of Thy-1-Fc increased their motility by ~ 25%, compared to 1% observed in their respective negative controls (Fig. 4g and h). The effect of Thy-1 on OMV-treated astrocytes was even greater than the ~ 10% increase in migration observed for astrocytes pretreated with TNF (Fig. 4h). Of note, under control conditions, i.e., in non-reactive astrocytes (white bars), Thy-1 did not affect astrocyte migration. Astrocytes treated with 3% FBS (positive control, light gray bar) migrated ~ 40% more than controls (white bars). Hence, treatment with *Hp* OMVs induces astrocyte reactivity, which increases the migratory response to Thy-1.

### Hp OMVs enhance NF-κB nuclear translocation and activity, which are required for β_3_ integrin and connexin 43 upregulation in astrocytes

**Fig. 5:**
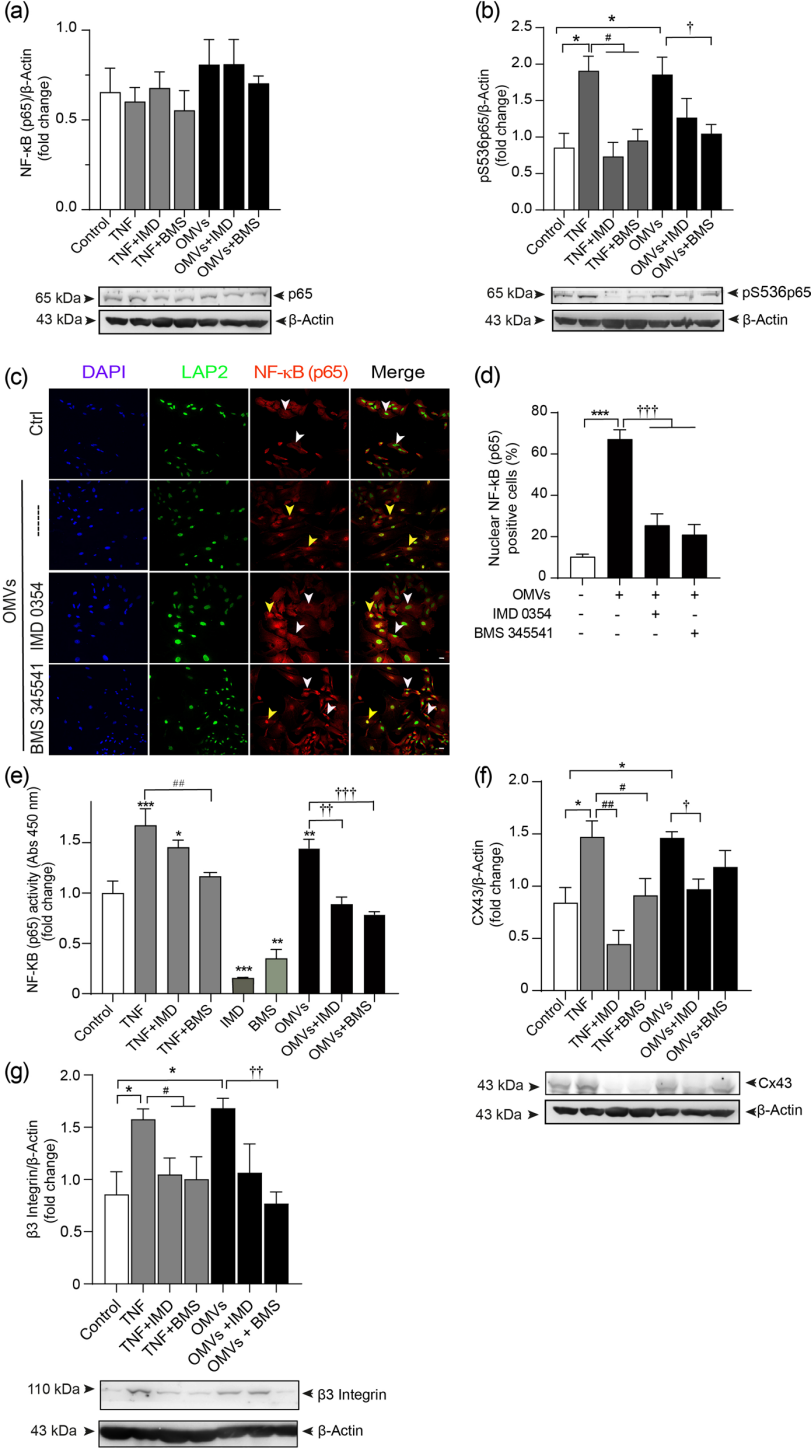
*Hp* OMVs-enhanced NF-κB nuclear translocation and activity are required for β_3_ integrin and connexin 43 upregulation in astrocytes. **a, b** Immunoblots of p65 NF-κB (**a**), (pS536p65) NF-κB (**b**) and β-actin (loading control) of astrocytes treated for 12 h with 2.5 µg/ml OMVs, with or without 1 µM IMD 0354 or 1 µM BMS 344551. Astrocytes incubated with TNF (10 ng/ml, 48 h) are the positive control samples. **c** Confocal microscopy of nuclear p65-positive cells (red), nuclear protein lamin-associated with peptide 2 (LAP-2) (green), and DAPI (blue) in astrocytes treated for 60 min with OMVs in the presence or absence of 1 μM IMD 0354 or 1 µM BMS 344551 (scale bar = 10 µm). The right panel is the merged image of LAP2, p65 and DAPI (scale bar = 10 µm). White and yellow arrows indicate the absence or presence of NF-κB in the nucleus, respectively. **d** Values in the graph represent the percentage of nuclear p65-positive cells (red nuclei) from the total nuclei (DAPI/LAP-2) (mean ± S.E.M.). The average number of cells was 100 astrocytes per condition, per experiment (*n* = 3). **e** ELISA assay of NF-κB DNA-binding activity in equal amounts of nuclear extracts obtained from astrocyte cultures treated for 60 min as indicated with *Hp* OMVs or TNF, with or without 1 μM IMD 0354 or 1 μM BMS 344551.** f, g** Immunoblots of connexin 43 (**f**), β_3_ integrin (**g**), and β-actin (loading control) of astrocytes treated with 2.5 µg/ml of *Hp* OMVs for 12 h, or with TNF (10 ng/ml, 48 h), ± 1 µM IMD 0354 or 1 μM BMS 344551. In all graphs, values are means ± S.E.M.; *n* = 3. *p < 0.05, **p < 0.01, ***p < 0.001, compared to control; ^#^*p* < 0.05, ^##^*p* < 0.01, compared to TNF; and ^†^*p* < 0.05, ^††^*p* < 0.01, ^†††^*p* < 0.001, compared to OMVs

Astrocytes of the A1 phenotype have a “destructive phenotype” under pro-inflammatory conditions, characterized by the activation of NF-κB [32, 34, 35]. To determine if NF-κB is involved in OMV-induced astrocyte reactivity, we first measured total levels of the NF-κB p65 subunit. We found that p65 levels were elevated at 6 h and remained elevated up to 72 h in DITNC1 cells treated with OMVs (Additional file 1: Figure S4), suggesting the participation of NF-κB in the reactivity process. In primary astrocytes, we then evaluated total p65 levels and its phosphorylated form (pS536p65), which indicates NF-κB activation [69]. The cells were also pre-incubated with the IMD 0354 (for IKKβ) or BMS 345541 (for IKKα and IKKβ) inhibitors. Total p65 did not increase in response to TNF or OMV treatment, but pS536p65 significantly increased in response to both stimuli (Fig. 5a and b, respectively). Phosphorylation of NF-κB was precluded by the two inhibitors (Fig. 5b), suggesting NF-κB activation in reactive astrocytes. We then evaluated nuclear translocation of NF-κB in astrocytes treated with 2.5 µg/ml of OMVs for 1 h, in the presence or absence of the IMD 0354 or BMS 345541 inhibitors. OMVs induced nuclear translocation of NF-κB (Fig. 5c) and more cells exhibited NF-κB (red) within the nucleus, stained with LAP2 (lamin protein marker, green). NF-κB appears yellowish in figures where red and green were merged (Fig. 5c). OMV-induced nuclear translocation of NF-κB was significantly elevated (~ 65%) compared with the ~ 10% of control astrocytes (Fig. 5d). Additionally, the IMD 0354 and BMS 345541 inhibitors prevented NF-κB translocation, and nuclear NF-κB levels were both significantly lower than those induced by OMVs, and more like controls (Fig. 5d). We then evaluated whether the nuclear translocation of NF-κB was associated with changes in its DNA-binding activity. We observed that astrocytes treated with TNF or OMVs increased NF-κB activity, an effect precluded when cells were co-incubated with BMS 345541 (Fig. 5e). However, co-incubation with IMD 0354 only prevented the increase of NF-κB activity when the cells were treated with OMVs, but not TNF (Fig. 5e). These results suggest that NF-κB transcriptional activity is induced in astrocytes treated with OMVs.

Connexin 43 and β_3_ integrin levels were upregulated in the positive control astrocytes (+ TNF), an effect that was prevented by the IMD 0354 and BMS 345541 inhibitors. The levels of these reactivity markers were also increased by OMV treatment, but did not increase with OMV treatment when the cells were pretreated with the IKK inhibitors (Fig. 5f and g), although their effect was not always significantly different, as found for TNF-treated cells (Fig. 5f and g). Thus, *Hp* OMVs induce a reactive phenotype (elevated connexin 43 and β_3_ integrin levels) in astrocytes through a mechanism dependent on NF-κB.

### Astrocytes rendered reactive by Hp OMVs promote neuronal damage

**Fig. 6:**
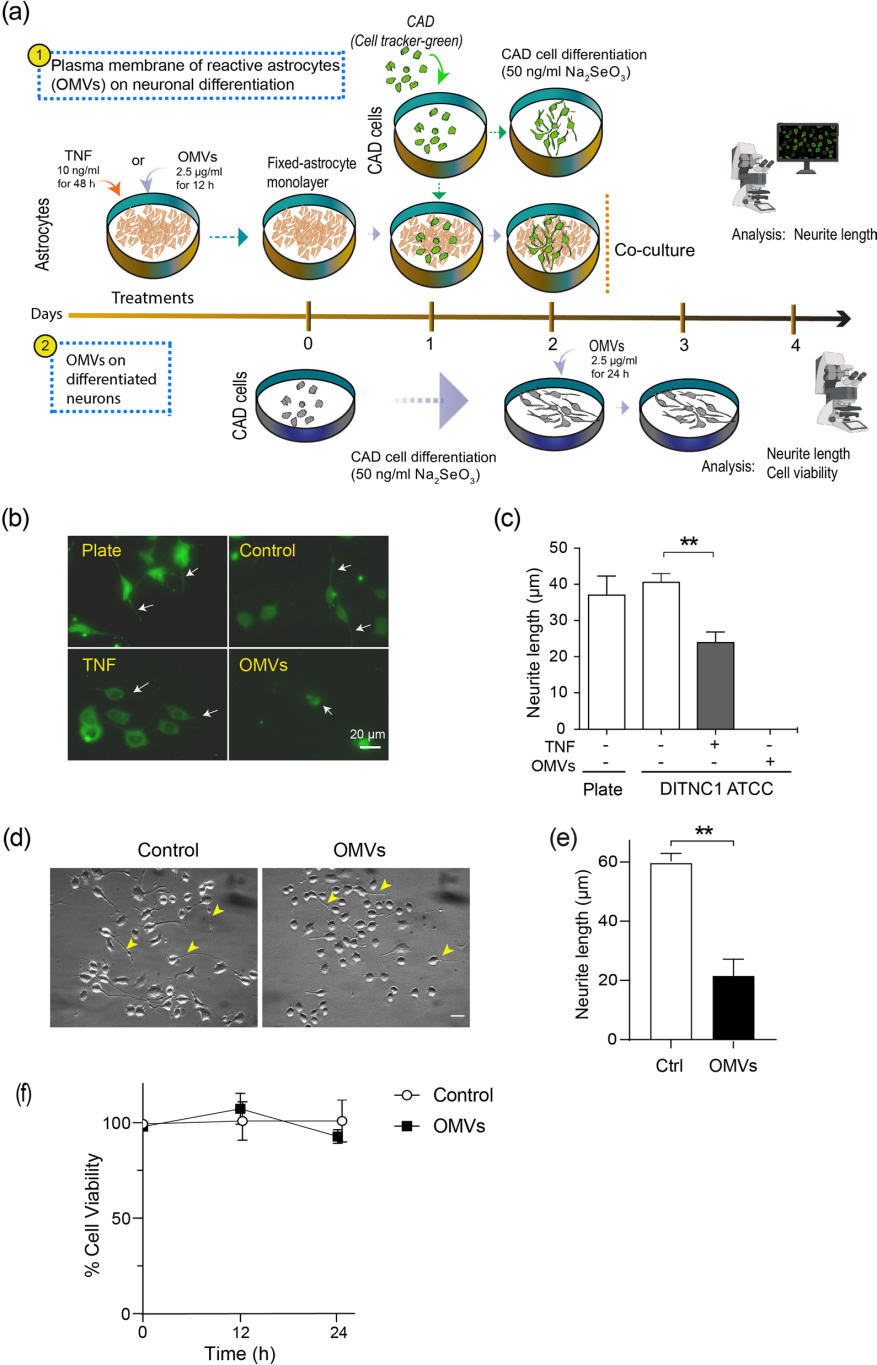
Astrocytes rendered reactive by *Hp* OMVs promote neuronal damage. **a** Experimental design to test the effect of (1) the plasma membrane of reactive astrocytes on neuronal differentiation, and (2) *Hp* OMVs on differentiated neurons. (1) Astrocytes were treated (with or without TNF or OMVs) and then fixed. CAD cells labeled with cell tracker green were cultured on a plate (control for CAD differentiation) or seeded and co-cultured on the fixed astrocyte monolayer for 24 h. (2) CAD cells differentiated for 48 h were treated with OMVs (2.5 μg/ml, 24 h) to explore the effect of OMVs on neuronal process retraction and cell viability. CAD cell morphological differentiation was estimated by measuring the length of neuronal processes using the NeuronJ plug-in of the NIH ImageJ Software. **b** Confocal microscopy of CAD cells (stained with cell tracker green) under the different experimental conditions. White arrows indicate neurites that extended on the tissue culture plate (plate), over untreated astrocytes (Control), or astrocytes treated with TNF or with OMVs (scale bar = 20 µm). **c** Quantification of neurite length (μm). The values in the graph are the means ± S.E.M.; *n* = 4, and **p < 0.01. We measured the neurite length of 50 neurons per condition, per experiment. **d** Phase-contrast microphotographs of CAD cells differentiated on the tissue culture plate without treatment (Control) or treated with OMVs (OMVs). Yellow arrowheads indicate neurites that extended on the tissue culture plate (scale bar = 20 µm). **e** Quantification of neurite length (µm) obtained from the images, as shown in **d**. Values are means ± S.E.M.; *n* = 4, and **p < 0.01. **f** MTS assay of differentiated CAD cells treated or not (Control) with OMVs for 12 or 24 h. The graph shows the percentage of viable cells

Communication between neurons and reactive astrocytes through the interaction between Thy-1 (neuron)/α_V_β_3_ integrin (astrocyte) is bidirectional. This interaction promotes astrocyte migration (Fig. 4g and h) and, in neurons, inhibits neurite extension and induces process retraction [55–57]. To evaluate the effect of OMV-treated reactive astrocytes on the inhibition of neurite outgrowth, astrocytes were treated with 2.5 µg/ml of OMVs for 12 h or TNF (10 ng/ml, 48 h), and fixed with paraformaldehyde. Fixation was included to assess the effects of astrocyte surface antigens rather than soluble factors released by the astrocytes. Then, CAD cells were seeded on glass bottom plates (positive differentiation control) or co-cultured on pretreated and fixed astrocytes to induce morphological differentiation of CAD cells by serum deprivation (Fig. 6a). To distinguish neurons from astrocytes in the co-cultures, neurons were labeled with Cell tracker green (Fig. 6b), a vital dye detectable for at least 72 h [70]. CAD cells co-cultured on TNF-pretreated astrocytes present a ~ 37% shorter neurite length than control astrocytes (untreated) or neuron monocultures (differentiated on plate) (Fig. 6b and c). However, CAD cells co-cultured on astrocytes pretreated with OMVs failed to differentiate (Fig. 6b and c). More dramatic changes were detected in astrocytes treated with OMVs, than with TNF. One interpretation is that OMVs remain attached to astrocytes and therefore, directly affect neurons. Hence, we tested whether the direct addition of OMVs onto differentiated neurons altered the retraction of neuronal processes and viability of CAD cells. CAD cells, differentiated for 48 h instead of 24 h to obtain longer neurites, were treated or not with OMVs for 24 h, fixed, and photographed using a bright field microscope. Neurite length was measured using the NeuronJ Software, as described [56]. Differentiated CAD cells treated with OMVs decreased neurite length by ~ 65% (Fig. 6d), compared to CAD controls (Fig. 6e). Treatment of CAD cells with OMVs did not alter CAD cell viability (Fig. 6f). Therefore, the dramatic effect of OMV-treated astrocytes, compared to TNF-treated ones, on neuronal differentiation could be due to the effects that OMVs exert on both astrocytes and neurons.

### Conditioned medium from astrocytes pretreated with Hp OMVs promotes CAD cell death and contains IFNγ

**Fig. 7:**
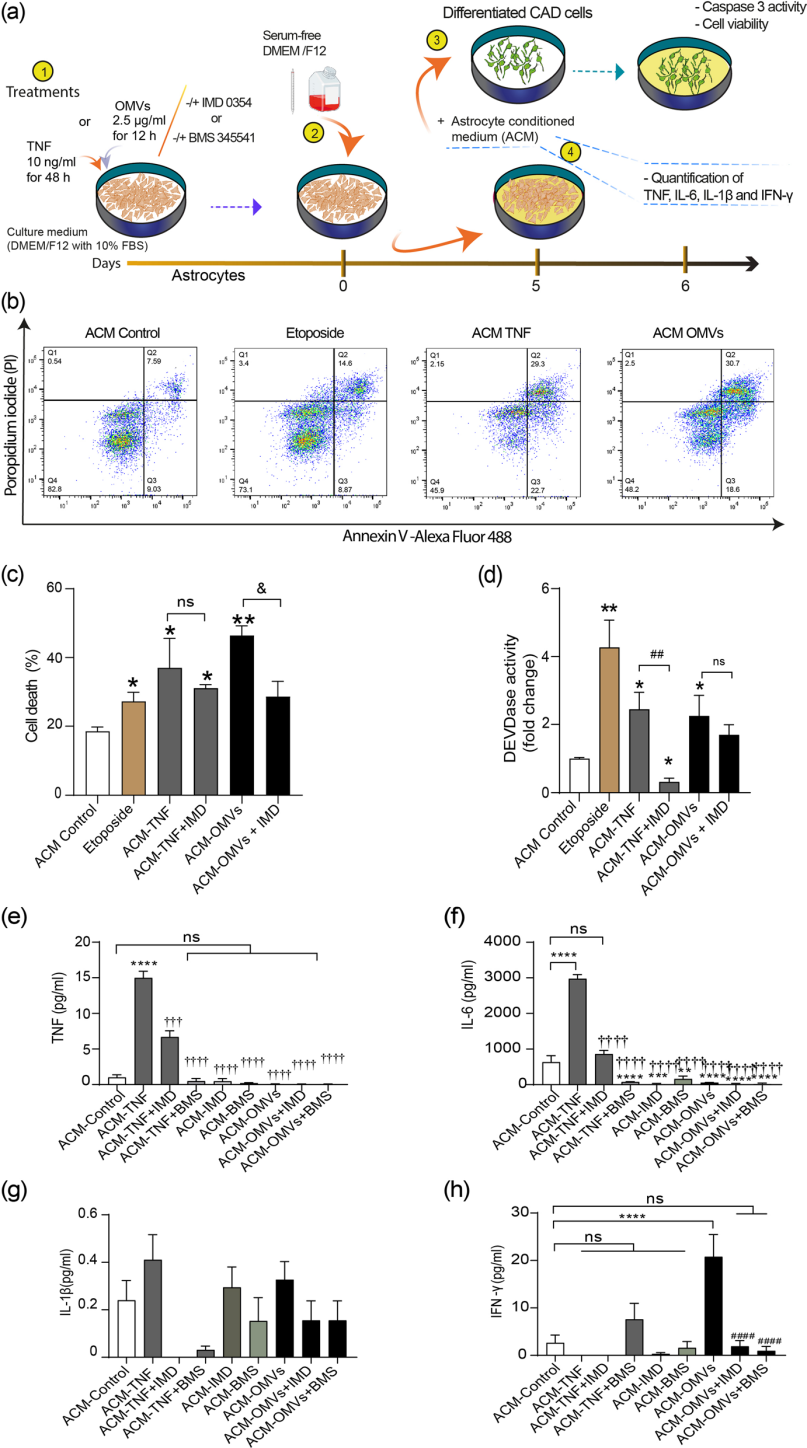
The conditioned medium from astrocytes pretreated with *Hp* OMVs promotes secretion of IFNγ and CAD cell death. **a** Representation of the different treatments: (1) untreated astrocytes, or treated with or without TNF or OMVs, in the presence or absence of NF-κB inhibitors; (2) after treatment, cells were washed and the medium was changed to SFM; (3) the collected astrocyte-conditioned medium (ACM) was added to differentiated CAD cells to evaluate cell viability and DEVDase activity (caspase 3); (4) collected ACM was used to quantify TNF, IL-6, IL-1β, and IFNγ using the MILLIPLEX RCYTMAG-65 K kit. **b** Dot plots of CAD cells using flow cytometry. CAD cells were harvested 24 h after treatment with ACM from astrocytes (ACM Control), etoposide (10 µM), or ACM obtained after treatment with TNF or OMVs for 24 h. Cells (10^5^) were incubated with AV conjugated to Alexa Fluor 488 and propidium iodide (PI). Dead cells are shown in the upper left quadrant (AV-negative but PI-positive, Q1); late or necrotic apoptotic cells, in the upper right quadrant (AV-positive and PI-positive, Q2); early apoptotic cells, in the lower right quadrant (AV-positive but PI-negative, Q3); and viable cells in the lower left quadrant (Q4). **c** The graph shows the percentage of the total dead cell populations (Q1 + Q2 + Q3) of differentiated CAD cells after the different treatments (etoposide or ACM ± IMD 0354 inhibitor pretreatment). Values in the graph are the means ± S.E.M.; *n* = 4; ns, non-significant, *p < 0.05, **p < 0.01, compared to ACM-control; and ^&^*p* < 0.05, compared to ACM-OMVs. **d** The graph shows the fold change of the DEVDase activity of CAD cells treated with 10 µM etoposide as a positive inducer of apoptosis, or of CAD cells treated with ACM from TNF-, or OMV-treated astrocytes ± 1 µM IMD 0354 inhibitor, compared to the ACM control. The results are expressed as the means ± S.E.M.; *n* = 3; ns, non-significant, *p < 0.05 and **p < 0.01, compared to the AMC-control; and ^##^*p* < 0.01, compared to AMC-TNF.** e–h** Quantification of the cytokines TNF (**e**), IL-6 (**f**), IL-1β (**g**) and IFNγ (**h**). Values in the graph are expressed as the means ± S.E.M; *n* = 3–4. **p < 0.01, ***p < 0.001, and ****p < 0.0001, compared to the ACM-control; ^####^*p* < 0.0001, compared to ACM-OMVs; ^†††^*p* < 0.001 and ^††††^*p* < 0.0001, compared to ACM-TNF

Considering that reactive astrocytes secrete unknown neurotoxic factors, and uptake and distribute brain metabolites [63, 71], we assessed whether the conditioned medium generated by astrocytes (ACM) previously treated with OMVs, affected neuronal viability. The ACM was obtained after different treatments and then added to differentiated CAD cells to test cell viability and caspase 3 activity (Fig. 7a). Treatment with 10 µM etoposide was used as a positive control for neuronal damage [50]. Externalization of plasma membrane phosphatidylserine was measured using fluorescently labeled AV to detect apoptotic cells, and cells were stained with propidium iodide (PI). As shown in Fig. 7b, in the ACM control-treated sample, 82% of cells were both AV- and PI-negative (viable). In the etoposide panel, the percentage of cells increased from 8 to 15%; these cells were positive for both AV and PI, and were found in the late stages of apoptosis, in which plasma membrane integrity is lost (secondary necrosis). Cells positive for AV were experimenting the early stages of apoptosis, with externalized phosphatidylserine, but an intact plasma membrane. Based on these histograms, when treated with ACM from TNF-treated astrocytes (ACM-TNF), 52% of cells were AV-positive, of which, 23% were in the early stages of apoptosis (only AV-positive), and 29% were in the late stages of apoptosis, moving towards secondary necrosis. Treatment with ACM from OMV-treated astrocytes yielded 50% of AV-positive cells, of which 19% were in early stages of apoptosis, and 31% were moving towards secondary necrosis. Cells undergoing necrosis become directly positive for PI only and represented 2–4% of the whole population of cells treated with either etoposide or ACM from TNF- or OMV-treated astrocytes (Fig. 7b). Figure 7c shows the results summary, where the percentage of cell death reflects the sum of cells in quadrants 1, 2, and 3. The effect of the ACM obtained from TNF- or OMV-treated astrocytes was significantly different from the control ACM. When the ACM was obtained from astrocytes treated with the IMD 0354 NF-κB inhibitor prior to TNF treatment, there was no effect of the IMD, and the values were like those of the ACM-TNF (Fig. 7c). On the contrary, when the ACM derived from astrocytes treated with IMD prior to the OMV treatment, the values differed significantly from those of the ACM OMVs samples. With respect to caspase 3 activity, differentiated CAD cells increased DVEDase activity more than twofold when treated with the ACM from OMV- or TNF-treated astrocytes, and fourfold when treated with 10 µM etoposide (Fig. 7d). When using the IMD 0354 inhibitor, the effect of the ACM from TNF-treated cells was lost, but not significantly reduced for ACM from astrocytes treated with OMVs. The treatment of astrocytes with OMVs leads to the release of soluble factors and these factors reduce neuronal viability in an NF-κB activation-dependent manner, while caspase 3 activation is NF-κB-independent.

Because NF-κB-target genes include TNF and other cytokines that may damage neurons, we assessed whether OMV-reactive astrocytes also produced pro-inflammatory cytokines. Astrocytes were treated with OMVs or with TNF, as a positive control, with or without 1 µM IMD 0354 or 1 µM BMS 345541 inhibitors. Then, the culture medium was removed and changed to a serum-free medium (SFM) to rule out participation of OMVs or TNF in the treatment. The ACM was obtained by incubating the astrocytes in the freshly added medium for 5 days. The collected ACM was filtered, and used to quantify pro-inflammatory cytokines (TNF, IL-6, IL-1β, and IFNγ) using a quantitative kit (Fig. 7a). TNF and IL-6 only increased in the ACM obtained from astrocytes pretreated with TNF, and significantly decreased when inhibited with IMD 0354 or BMS 345541 (Fig. 7e and f). Levels of IL-1β in the ACM did not change significantly when obtained from astrocytes treated under the different experimental conditions (Fig. 7g). Only IFNγ increased in the ACM obtained from reactive astrocytes pretreated with OMVs, and its secretion was reduced to control levels with IMD 0354 or BMS 345541 treatment (Fig. 7h). Our results suggest that *Hp* OMV-reactive astrocytes release IFNγ into the extracellular medium, which depends on NF-κB signaling and activity.

## Discussion

Our study revealed that following systemic (tail vein injection) and oral administration, *Hp* OMVs can access the mouse brain and alter astrocytes and neurons in vivo. OMVs induce astrocyte reactivity, and exposure to either OMVs or OMV-induced reactive astrocytes triggers retraction of neuronal processes and inhibits neurite outgrowth. OMV-treated astrocytes exhibit NF-κB activation and IFNγ secretion, and the astrocyte-conditioned medium harms neurons in vitro. To our knowledge, this is the first study describing that *Hp* OMVs trigger extra-gastric systemic effects in neurons and astrocytes.

The average size of *Hp* OMVs is 20–450 nm [18, 45]. Here, the OMVs from the 60190 strain averaged a size of 126.5 ± 6.5 nm. *Hp* OMVs from the 26695 strain have a mean diameter of 120.2 ± 39.1 nm, and OMVs from patient-derived strains measure 89–127 nm [72]. Although heterogeneous in size, vesicles derived from various *Hp* strains, including patient-derived strains, are similar in size to those of *Hp* 60190. Furthermore, the OMVs isolated by the method described in the present study are less heterogeneous than those obtained via a sucrose gradient separation, which can have diameters as large as 450 nm, as previously reported [18].

The surface of OMVs is composed of a phospholipid bilayer with an outer layer of LPS and various bacterial outer membrane proteins involved in inflammation. These proteins are essential in the colonization, adherence, and virulence of *Hp* [73]. Our proteomics results (Fig. 1e) revealed that the protein content of OMVs is similar to that of the parental bacterial pellet, although enriched in specific proteins. These results suggest that a selection process is involved in the biogenesis and/or release of these vesicles. However, the molecular mechanisms describing the biogenesis of OMVs from Gram-negative bacteria and, particularly *Hp,* have not been fully elucidated yet [74–76].

An accumulation of misfolded proteins in the bacterial periplasm increases the physical distance between the outer membrane and its peptidoglycans, thus favoring the formation and release of more OMVs to the extracellular space [77]. Even though the contents of OMVs may vary among different preparations and time points of bacterial cultures [78], our proteomic results revealed high levels of similarly represented proteins in at least 3 different OMV preparations. In addition, several of these enriched proteins in OMVs have been previously detected in *Hp* OMVs of various *Hp* strains, including those from clinical isolates [79]. We found that OMVs are enriched in the 60-kDa chaperonin protein (GroEL) and urease. GroEL promotes protein folding and misfolding in various bacteria [80], and has also been identified by proteomics studies in OMVs from other bacteria, such as *Acinetobacter baumannii* [81]. GroEL also binds to the UreA subunit, another virulence factor highly represented in our proteomics analysis, and participates in urease maturation in the cytosol and on the bacterial surface [82]. We suggest that GroEL supports the production of OMVs, as well as the functionality of urease, and perhaps of other cargo proteins in OMVs.

As reported, bacterial OMVs are related to brain disorders and memory. Injection of OMVs from the microbiome of Alzheimer’s patients into the bloodstream of mice induces loss of spatial learning and memory, activation of microglia and astrocytes, increased pro-inflammatory cytokines in the brain, and increased permeability of the BBB [28]. OMVs from *Paenalcaligenes hominis* can also reach the mouse brain either via the blood circulation or the vagus nerve, causing cognitive disorders like those of Alzheimer’s disease [83]. Daily intraperitoneal injections with *Hp* culture filtrates induce loss of spatial memory and learning in rats [84]. Also, mice that had been infected with *Hp* for 5 months [14] or *H. felis* for 18 months show neuroinflammation [15]. In the present study, we show that systemic injection of a single dose of *Hp* OMVs results in vesicles reaching the brain, astrocyte reactivity and neuronal damage. Our evidence suggests that bacteria might affect the brain by releasing OMVs to the bloodstream. However, how *Hp* OMVs access the brain and whether the effects are similar for OMVs from different strains, and/or isogenic mutant strains with or without key virulence factors, are still being investigated.

Another unanswered question is which are the astrocyte receptors responsible for the effects caused by the *Hp* OMVs. Astrocytes recognize pathogens through Toll-like receptors (TLRs) [85]. TLR2 is upregulated by TNF and NF-κB-dependent pathways [86], suggesting that reactive astrocytes may recognize many bacteria or OMVs. Our results show that OMV-reactive astrocytes increase IFNγ production through a NF-κB-dependent pathway. Therefore, OMVs may activate upstream signaling receptors that increase IFNγ expression, such as TLRs. Here, TLR2 might act as a receptor for OMVs, since *Hp* urease induces HIF-1α via a TLR2-dependent mechanism in gastric cells [87]. Even though the receptors for *Hp* OMVs in astrocytes are still unknown, TLR2 is a plausible receptor for urease, present in OMVs.

The high levels of urease in *Hp* OMVs were surprising (Fig. 1). *Hp* urease not only helps bacteria survive in the stomach by generating ammonia, which neutralizes the acidic pH, but can become toxic if its production is excessive [88]. The resulting hyperammonemia could induce changes in the permeability of the BBB given its effect in endothelial cells [87] and aid in shuffling urease into the brain. Whether *Hp* urease crosses the BBB as a soluble molecule or in the form of microvesicles has not been reported; however, our results indicating the enriched presence of urease in *Hp* OMVs tends to favor the idea of neurotoxicity triggered by hyperammonemia [90].

Further studies are necessary to identify the molecular basis of the interactions between the bacterial vesicles and astrocytes and the effects of Hp OMVs on these cells.

Reactive A1-type astrocytes are neurotoxic, their activation involves NF-κB-dependent pathways, and they have been implicated in several neurodegenerative diseases [92]. As described, *Hp* OMVs activate NF-κB, increase the expression of intermediate filament proteins (GFAP and vimentin), hemichannels (Cx43), membrane proteins (α_V_β_3_ integrin), and IFNγ. Since the ACM from OMV-reactive astrocytes also has a detrimental effect on neurons, it is likely that OMVs induce a reactive “A1” phenotype in astrocytes. Accordingly, IFNγ and IFNγR deficiency in mice prevents the activation of the A1 reactive astrocytes and restores neurogenesis and cognitive function [93]. Since OMVs induce astrocyte reactivity and reactive astrocytes damage neurons, bacterial endotoxins and/or factors secreted by astrocytes could therefore cause or contribute to neurodegeneration.

The inhibition of neurite outgrowth in CAD cells produced by OMV-activated astrocytes could be caused by OMVs that directly bind to the surface of astrocytes or by molecular changes occurring on the surface of reactive astrocytes. Previous in vitro studies demonstrated that the in trans interaction between neuronal Thy-1 and the astrocytic α_V_β_3_ integrin, which is upregulated in reactive astrocytes, inhibits neurite outgrowth and promotes retraction of neuronal processes [55, 57]. Importantly, the present study revealed that α_V_β_3_ integrin was overexpressed in astrocytes treated with *Hp* OMVs, and that the extension of neuronal processes was inhibited. Therefore, the effects of OMVs on neurons may be attributable to the Thy-1–α_V_β_3_ integrin interaction between reactive astrocytes and neurons. Of note, OMVs also cause retraction of neuronal processes, but without affecting cell viability (Fig. 6). Therefore, OMVs directly bound to astrocytes could contribute to the effect of astrocytes on neuronal cells.

On the other hand, differentiated CAD cells died when treated with the ACM obtained from astrocytes pretreated with TNF or OMVs. However, neuronal cell death decreased after treatment with the ACM from OMVs-treated astrocytes obtained in the presence of a NF-κB inhibitor. Additionally, similar treatment with ACM from OMV-treated astrocytes led to IFNγ secretion, which was also inhibited when the ACM was obtained from astrocytes pretreated with the NF-κB inhibitor. Therefore, we speculate that astrocytes treated with OMVs release neurotoxic factors (perhaps IFNγ), dependent on NF-κB signaling, and such factors induce neuronal death in vitro. Noteworthy, our results do not discard the possibility that ACM from OMV-treated astrocytes could still contain free OMVs, which would account for the damaging effect on neurons; however, results shown in Fig. 6e and f indicate that although OMVs could induce neurite retraction, they do not kill neurons.

## Conclusions

In this study, we present evidence that connects the effects of *Hp* OMVs in the brain of mice with the genesis of inflammatory conditions in vivo. Furthermore, we provide in vitro evidence that OMVs induce astrocyte reactivity through NF-κB-dependent signaling, secretion of IFNγ and possibly, other unknown toxic factors. Additionally, we show that the OMVs, the surface molecules of OMV-reactive astrocytes, and the ACM produced by such reactive astrocytes provoke neuronal damage, such as neurite retraction, and ultimately, increase neuronal death. Therefore, we propose that OMVs represent nanocarriers of *Hp* virulence factors to the CNS and thus, serve as vectors for the transmission of information from the gastric microbiota to the brain, where they contribute to some of the reported systemic effects of *Hp*. Importantly, our evidence indicating the neuroinflammatory effect of *Hp*-OMVs in mice injected with these vesicles should also encourage studies for early *Hp* detection and treatment to eradicate this pathogen.

## Supplementary Information

**Additional file 1. Table S1.** Identification of proteins with unique peptides in Helicobacter pylori (*Hp*) 60190 OMVs. The results of the LC–MS/MS analysis and the search using the *Hp* UniProtKB database (UP000000429.fasta) allowed to identify the total protein content of OMVs and the parental bacteria *Hp* 60190. The table shows the protein ID and the average number of unique peptides of proteins found in OMVs. **Figure S1.** Evaluation of the dose-response of the reactivity markers in DITNC1 ATCC astrocytes treated with different concentrations of OMVs from Helicobacter pylori (*Hp*) 60190. DITNC1 ATCC cells were incubated in the absence or presence of OMVs (1.25 to 20 µg/ml) from *Hp* 60190, at 37 °C for 24 h. As a positive reactivity control, the astrocytes were incubated with TNF (10 ng/ml, 48 h). After treatment, whole cell lysates were evaluated by immunoblot analysis of total connexin 43 (A), β3 integrin (B), GFAP (C), and vimentin (D), normalized to β-actin. Values in the graphs were obtained by averaging the immune-specific band intensity normalized to β-actin from 3 independent experiments (mean ± S.E.M). *p < 0.05 and **p < 0.01, compared to controls (Ctrl). **Figure S2.** Dose-response of the reactivity markers in DITNC1 ATCC astrocytes treated with 2.5 µg/ml of Helicobacter pylori (*Hp*) OMVs at different times. DITNC1 ATCC cells were incubated in the absence or presence of 2.5 µg/ml of *Hp* 60190 OMVs at 37 °C, from 3 to 72 h. As a positive reactivity control, the astrocytes were incubated with TNF (10 ng/ml, 48 h). After treatment, whole cell lysates were evaluated by immunoblot analysis of total connexin 43 (A), β3 integrin (B), GFAP (C), and vimentin (D), normalized to β-actin. Values in the graphs were obtained by averaging the immune-specific band intensity, normalized to β-actin, from 3 independent experiments (mean ± S.E.M). *p < 0,05, **p < 0.01, and ***p < 0.001, compared to controls (Ctrl). **Figure S3.** Effect of Helicobacter pylori (*Hp*) OMVs on astrocyte cell viability. Primary astrocytes (post 17 days in vitro) were incubated in the absence or presence of 2.5 µg/ml of OMVs for 12 h. Cell viability was evaluated with the Trypan blue assay in a Neubauer chamber. Values in the graph represent the average of the percentage of negative trypan blue cells from 3 independent experiments (mean ± S.E.M.). **Figure S4.** Effect of Helicobacter pylori (*Hp*) OMVs on p65 NF-ĸB subunit protein levels in DITNC1 ATCC astrocytes, following different times of exposure. DITNC1 ATCC cells were incubated in the absence or presence of 2.5 µg/ml of OMVs from 3 to 72 h, at 37 °C. As a positive reactivity control, the astrocytes were incubated with TNF (10 ng/ml, 48 h). After treatment, whole cell lysates were evaluated by immunoblot analysis of p65 NF-ĸB total levels. Values in the graph were obtained by averaging the immune-specific band intensity, normalized to β-actin, from 3 independent experiments (mean ± S.E.M). *p < 0.05, **p < 0.01, and ***p < 0.001, compared to controls (Ctrl). **Table S2.** Research Resource IdentifiersList of reagents, antibodies, inhibitors, kits, experimental models used in this study. Tee catalog number is included when available.

## Data Availability

Any additional information required to reanalyze the data reported in this paper are available from the authors upon request.

## Abbreviations

ACM: Astrocyte-conditioned medium
AFC: Amino-4-trifluoromethylcoumarin
AV: Annexin V
BBB: Blood–brain barrier
CAD: Cath.-a-differentiated
CNS: Central nervous system
Cx43: Connexin 43
DiR: 1,1′-Dioctadecyl-3,3,3′,3′-tetramethylindotricarbocyanine iodide
FBS: Fetal bovine serum
GFAP: Glial fibrillar acidic protein
*Hp*: *Helicobacter pylori*
IFNγ: Interferon gamma
IKK: Inhibitory-κB kinase
LAP2: Lamin-associated polypeptide 2
LC–MS/MS: Liquid chromatography–mass spectrometry
LPS: Lipopolysaccharide
NF-κB: Nuclear factor *kappa* B
OMVs: Outer membrane vesicles
PBS: Phosphate buffer saline
PI: Propidium iodide
S.E.M.: Standard error of the mean
SFM: Serum-free medium
Thy-1: Thymocyte differentiation antigen 1
TLRs: Toll-like receptors
TNF: Tumor necrosis factor
