# Supplementary material for: Helicobacter pylori outer membrane vesicles induce astrocyte reactivity through nuclear factor-κappa B activation and cause neuronal damage in vivo in a murine model
Source: J Neuroinflammation. 2023 Mar 9;20:66. doi: 10.1186/s12974-023-02728-7 (PMC9996972; doi:10.1186/s12974-023-02728-7)
Supplement: Supplementary file 1 — Additional file 1. Table S1. Identification of proteins with unique peptides in Helicobacter pylori (Hp) 60190 OMVs. The results of the LC–MS/MS analysis and the search using the Hp UniProtKB database (UP000000429.fasta) allowed to identify the total protein content of OMVs and the parental bacteria Hp 60190. The table shows the protein ID and the average number of unique peptides of proteins found in OMVs. Figure S1. Evaluation of the dose-response of the reactivity markers in DITNC1 ATCC astrocytes treated with different concentrations of OMVs from Helicobacter pylori (Hp) 60190. DITNC1 ATCC cells were incubated in the absence or presence of OMVs (1.25 to 20 µg/ml) from Hp 60190, at 37 °C for 24 h. As a positive reactivity control, the astrocytes were incubated with TNF (10 ng/ml, 48 h). After treatment, whole cell lysates were evaluated by immunoblot analysis of total connexin 43 (A), β3 integrin (B), GFAP (C), and vimentin (D), normalized to β-actin. Values in the graphs were obtained by averaging the immune-specific band intensity normalized to β-actin from 3 independent experiments (mean ± S.E.M). *p < 0.05 and **p < 0.01, compared to controls (Ctrl). Figure S2. Dose-response of the reactivity markers in DITNC1 ATCC astrocytes treated with 2.5 µg/ml of Helicobacter pylori (Hp) OMVs at different times. DITNC1 ATCC cells were incubated in the absence or presence of 2.5 µg/ml of Hp 60190 OMVs at 37 °C, from 3 to 72 h. As a positive reactivity control, the astrocytes were incubated with TNF (10 ng/ml, 48 h). After treatment, whole cell lysates were evaluated by immunoblot analysis of total connexin 43 (A), β3 integrin (B), GFAP (C), and vimentin (D), normalized to β-actin. Values in the graphs were obtained by averaging the immune-specific band intensity, normalized to β-actin, from 3 independent experiments (mean ± S.E.M). *p < 0,05, **p < 0.01, and ***p < 0.001, compared to controls (Ctrl). Figure S3. Effect of Helicobacter pylori (Hp) OMVs on astrocyte cell via [file 12974_2023_2728_MOESM1_ESM.docx]

**Supplementary Information**

***Helicobacter pylori* outer membrane vesicles induce astrocyte reactivity through Nuclear Factor-κappa B activation and cause neuronal damage *in vivo* in a murine model**

Running title: *H. pylori* vesicles and astrocyte reactivity

Esteban Palacios^1,2,3^, Lorena Lobos-González^3,6^, Simón Guerrero^3,4,7^, Marcelo J. Kogan^3,4^, Baohai Shao^5^, Jay W. Heinecke^5^, Andrew F.G. Quest^2,3^, Lisette Leyton^2,3,^* and Manuel Valenzuela-Valderrama^1,3,^*

**Affiliations**

^1^Laboratorio de Microbiología Celular, Instituto de Investigación y Postgrado, Facultad de Ciencias de la Salud, Universidad Central de Chile, Santiago 8330546, Chile.

^2^ Laboratory of Cellular Communication, Center for Studies on Exercise Metabolism and Cancer (CEMC), Institute of Biomedical Sciences (ICBM). Facultad de Medicina, Universidad de Chile, Santiago 8380453, Chile.

^3^ Advanced Center for Chronic Diseases (ACCDiS), Facultad de Ciencias Químicas y Farmacéuticas, Universidad de Chile, Santiago 8380494, Chile.

^4^ Departamento de Química Farmacológica y Toxicológica, Facultad de Ciencias Químicas y Farmacéuticas, Universidad de Chile, Santiago 8380494, Chile.

^5^ Division of Metabolism, Endocrinology and Nutrition, University of Washington, Seattle, WA 98195-8055, USA.

^6^ Centro de Medicina Regenerativa, Facultad de Medicina, Universidad del Desarrollo-Clínica Alemana, Santiago 7590943, Chile.

^7^ Facultad de Medicina, Universidad de Atacama,1533601 Copiapó, Chile.

*Corresponding Authors: lleyton@uchile.cl (LL) and manuel.valenzuela@ucentral.cl (MVV)


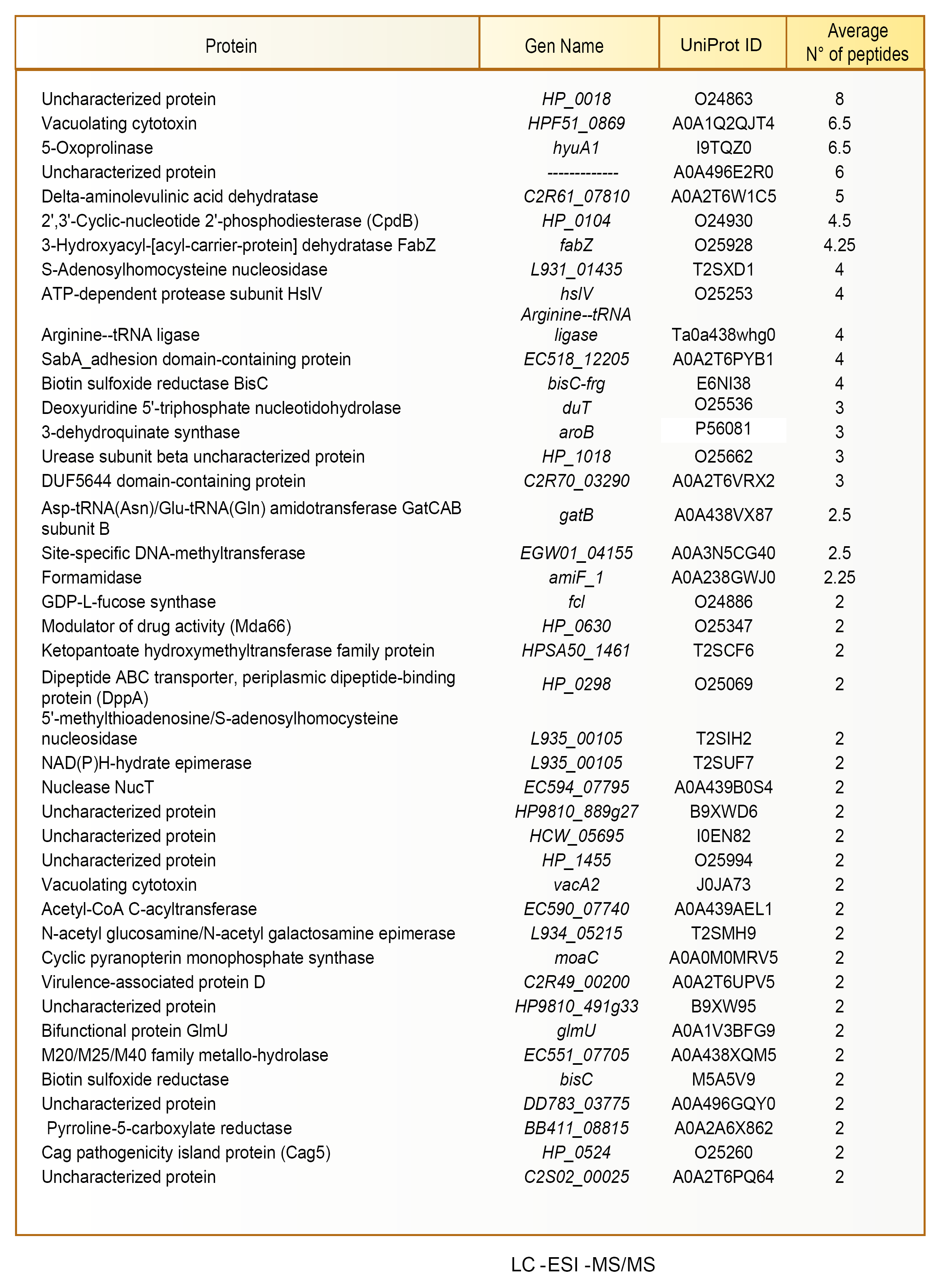


**Table S1: Identification of proteins with unique peptides in** ***Helicobacter pylori* *(Hp)* 60190 OMVs.** The results of the LC–MS/MS analysis and the search using the *Hp* UniProtKB database (UP000000429.fasta) allowed to identify the total protein content of OMVs and the parental bacteria *Hp* 60190. The table shows the protein ID and the average number of unique peptides of proteins found in OMVs.

**
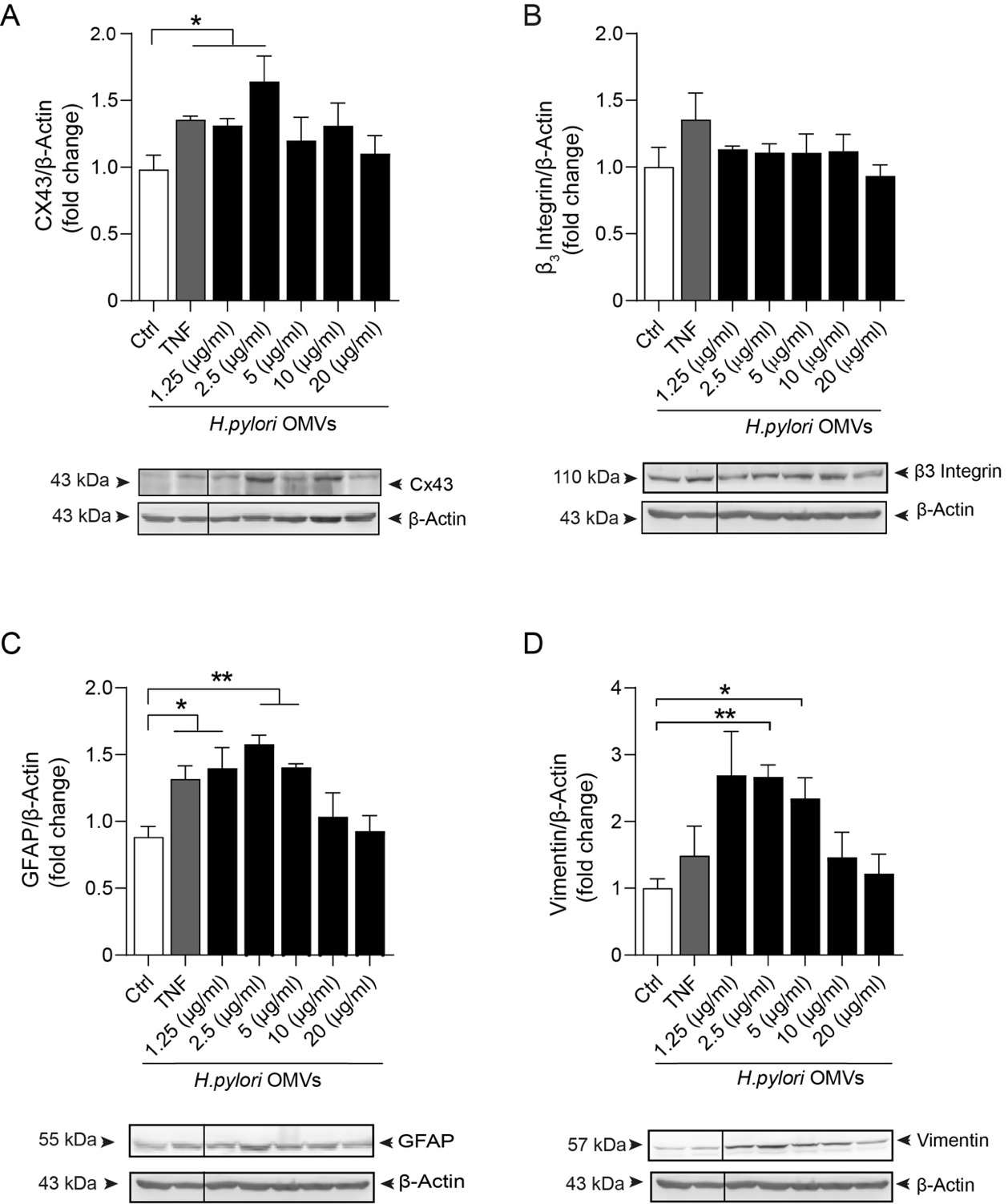
**

**Figure S1. Evaluation of the dose-response of the reactivity markers in DITNC1 ATCC astrocytes treated with different concentrations of OMVs from *Helicobacter pylori (Hp)* 60190**. DITNC1 ATCC cells were incubated in the absence or presence of OMVs (1.25 to 20 µg/ml) from *Hp* 60190, at 37 °C for 24 h. As a positive reactivity control, the astrocytes were incubated with TNF (10 ng/ml, 48 h). After treatment, whole cell lysates were evaluated by immunoblot analysis of total connexin 43 (A), β_3_ integrin (B), GFAP (C), and vimentin (D), normalized to β-actin. Values in the graphs were obtained by averaging the immune-specific band intensity normalized to β-actin from 3 independent experiments (mean ± S.E.M). **p* < 0.05 and ***p* < 0.01, compared to controls (Ctrl).

**
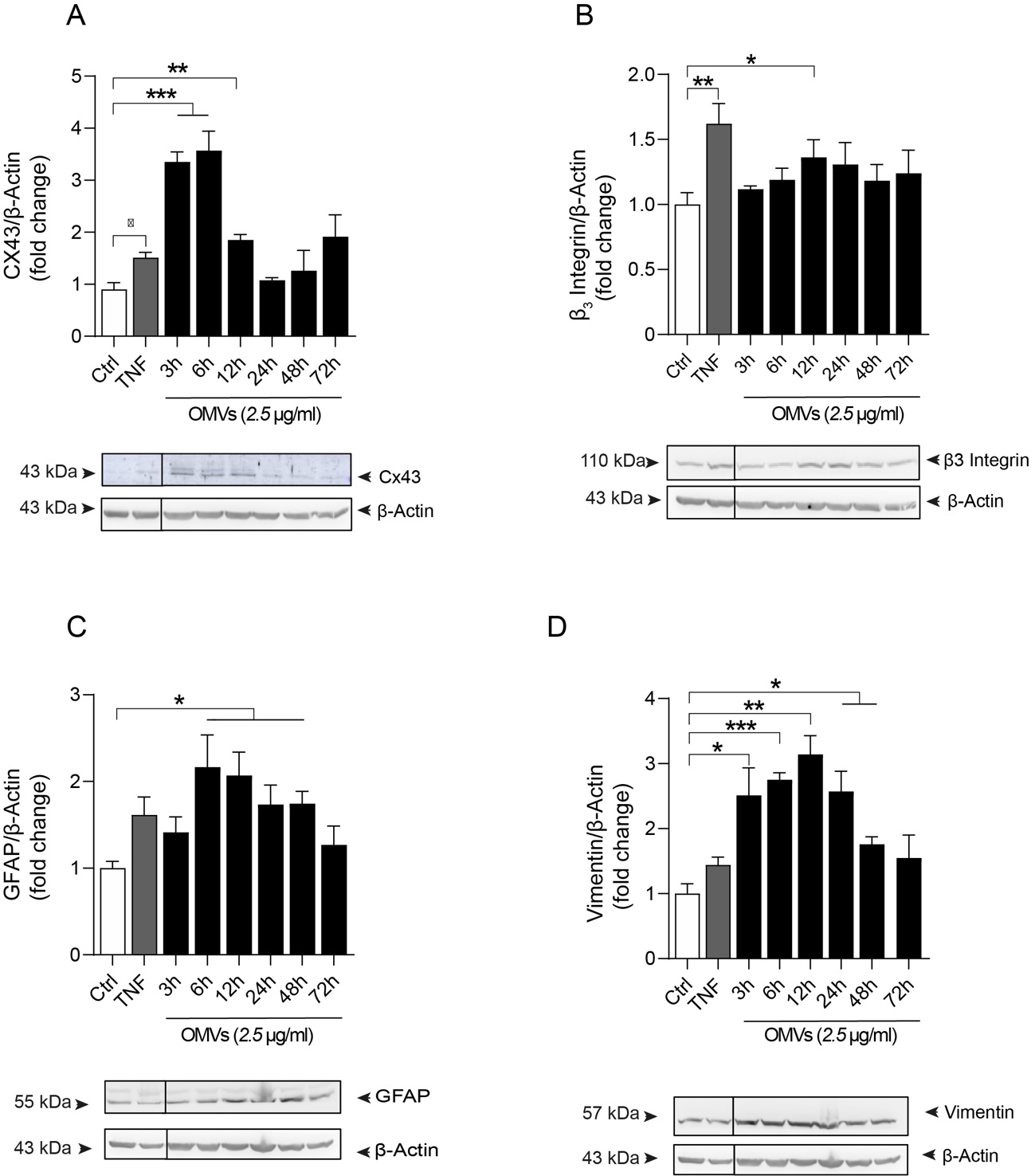
**

**Figure S2. Dose-response of the reactivity markers in DITNC1 ATCC astrocytes treated with 2.5 µg/ml of *Helicobacter pylori (Hp)* OMVs at different times**. DITNC1 ATCC cells were incubated in the absence or presence of 2.5 µg/ml of *Hp* 60190 OMVs at 37 °C, from 3 to 72 h. As a positive reactivity control, the astrocytes were incubated with TNF (10 ng/ml, 48 h). After treatment, whole cell lysates were evaluated by immunoblot analysis of total connexin 43 (A), β_3_ integrin (B), GFAP (C), and vimentin (D), normalized to β-actin. Values in the graphs were obtained by averaging the immune-specific band intensity, normalized to β-actin, from 3 independent experiments (mean ± S.E.M). **p* < 0,05, ***p* < 0.01, and ****p* < 0.001, compared to controls (Ctrl).


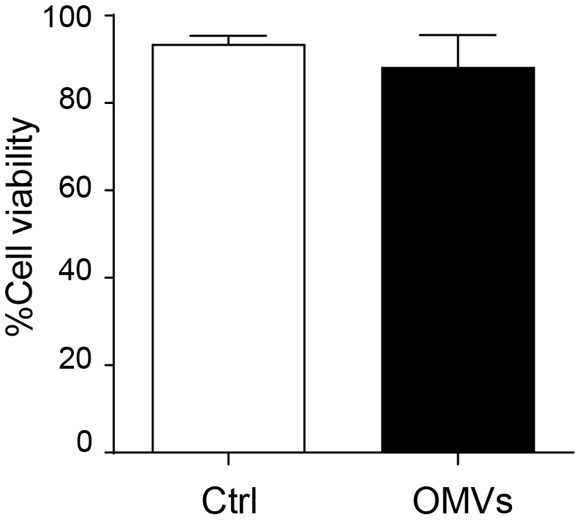


**Figure S3. Effect of *Helicobacter pylori (Hp)* OMVs on astrocyte cell viability**. Primary astrocytes (post 17 days *in vitro*) were incubated in the absence or presence of 2.5 µg/ml of OMVs for 12 h. Cell viability was evaluated with the Trypan blue assay in a Neubauer chamber. Values in the graph represent the average of the percentage of negative trypan blue cells from 3 independent experiments (mean ± S.E.M.).


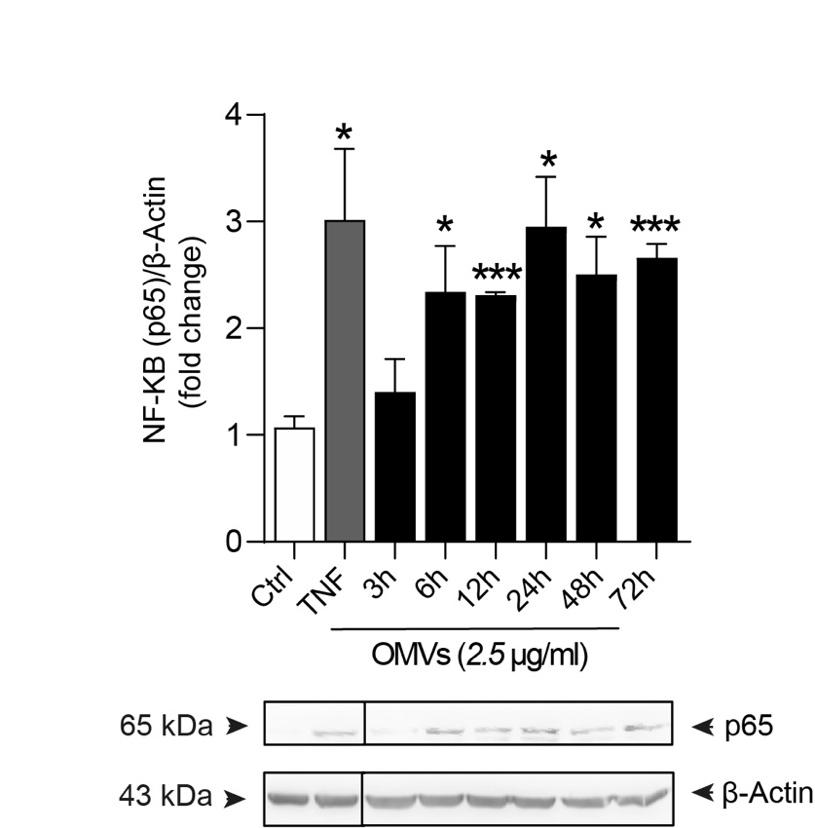


**Figure S4. Effect of *Helicobacter pylori (Hp)* OMVs on p65 NF-ĸB subunit protein levels in DITNC1 ATCC astrocytes, following different times of exposure**. DITNC1 ATCC cells were incubated in the absence or presence of 2.5 µg/ml of OMVs from 3 to 72 h, at 37 °C. As a positive reactivity control, the astrocytes were incubated with TNF (10 ng/ml, 48 h). After treatment, whole cell lysates were evaluated by immunoblot analysis of p65 NF-ĸB total levels. Values in the graph were obtained by averaging the immune-specific band intensity, normalized to β-actin, from 3 independent experiments (mean ± S.E.M). **p* < 0.05, ***p* < 0.01, and ****p* < 0.001, compared to controls (Ctrl).

***Table 2. Research Resource Identifiers***. List of reagents, antibodies, inhibitors, kits, experimental models used in this study. Tee catalog number is included when available.

| REAGENT or RESOURCE | SOURCE | IDENTIFIER |
| --- | --- | --- |
| Antibodies | | |
| Mouse monoclonal anti-GFAP | Sigma-Aldrich | Cat# G3893; [AB_477010](http://antibodyregistry.org/AB_477010) |
| Mouse monoclonal anti-β-actin | Sigma-Aldrich | Cat# A5316; [AB_476743](http://antibodyregistry.org/AB_476743) |
| Mouse monoclonal anti-vimentin | Santa Cruz | Cat# SC-66002; [AB_1131285](http://antibodyregistry.org/AB_1131285) |
| Rabbit polyclonal anti-connexin 43 | Santa Cruz | Cat# SC-9059; [AB_638640](http://antibodyregistry.org/AB_638640) |
| Rabbit polyclonal anti-β_3_ integrin | Millipore | Cat# AB2984; [AB_10806204](http://antibodyregistry.org/AB_10806204) |
| Rabbit monoclonal anti-NF-κB (p65) | Cell Signaling | Cat# 8242; [AB_10859369](http://antibodyregistry.org/AB_10859369) |
| Rabbit monoclonal anti-pS536p65 | Cell Signaling | Cat# 3033; [AB_331284](http://antibodyregistry.org/AB_331284) |
| Mouse monoclonal anti-Thy-1/CD90 | BD Biosciences | Cat# 554894; [AB_395585](http://antibodyregistry.org/AB_395585) |
| Mouse monoclonal anti-βIII tubulin | Promega | Cat# G7121; [AB_430874](http://antibodyregistry.org/AB_430874) |
| Mouse monoclonal anti-LAP2 | BD Biosciences | Cat# 611000; [AB_398313](http://antibodyregistry.org/AB_398313) |
| Goat- anti-mouse IgG (H+L) polyclonal antibody (HRP) | SeraCare KPL | Cat# 074-1806; [AB_2891080](http://antibodyregistry.org/AB_2891080) |
| Goat anti-rabbit IgG (H+L) polyclonal antibody (HRP) | SeraCare KPL | Cat# 074-1506; AB_2721169 |
| Goat anti-mouse IgG, Alexa 488 | Molecular Probes | Cat# A-11029; AB_138404 |
| Goat anti-rabbit IgG; Alexa 546 | Molecular Probes | Cat# A-11010; AB_2534077 |
| Bacterial and virus strains | | |
| *Helicobacter pylori* 60190 (cag PAI+, VacA s1m1) | ATCC | ATCC 49503 |
|  |  |  |
| Biological samples |  |  |
|  |  |  |
| Chemicals, peptides, and recombinant proteins | | |
| Thy-1-Fc | Department of Biochemistry, University of Lausanne, Switzerland | Dr. Pascal Schneider |
| TRAIL-R2-Fc | Department of Biochemistry, University of Lausanne, Switzerland | Dr. Pascal Schneider |
| Fc-TNF | Department of Biochemistry, University of Lausanne, Switzerland | Dr. Pascal Schneider |
| Invitrogen™ Exosome Spin Columns (MW 3000) | Invitrogen | Cat# 4484449 |
| Vitox | Thermo Fisher Scientific | Cat# SR0090A |
| Amicon Ultra 15 ml-100 kDa | Sigma-Aldrich | Cat# UFC910008 |
| Trypan Blue Solution | Gibco | Cat# 11538886 |
| Granulated agar | Difco | Cat# 214530 |
| Complete mini protease inhibitor cocktail tablets | Roche | Cat# 0469312401 |
| β-cyclodextrin | Sigma-Aldrich | Cat# C4767 |
| IMD 0354 | Sigma-Aldrich | Cat# I3159 |
| BMS 345541 | Sigma-Aldrich | Cat# B9935 |
| Glycine | Sigma-Aldrich | Cat# G7126 |
| Paraformaldehyde | Sigma-Aldrich | Cat# P6148 |
| DAPI | Invitrogen | Cat#: 62248 |
| Brucella broth | Thermo Fisher Scientific | Cat# R452662 |
| Dent | Thermo Fisher Scientific | Cat# SR0147E |
| Acrylamide/Bis acrylamide, 40% solution | Bio Rad | Cat# 1610146 |
| Sodium selenite | Sigma-Aldrich | Cat# S5261 |
| Cell Tracker Green CMFDA | Thermo Fisher Scientific | Cat# C2925 |
| DiR | Thermo Fisher Scientific | Cat# D12731 |
| Fetal bovine serum (FBS) | Corning | Cat# 35-010-CU |
| Etoposide | Sigma-Aldrich | Cat# 33419-42-0 |
| AFC | Enzo | Cat# ALX-260-032-M005 |
| RPMI | GIBCO | Cat# 23400-013 |
| DMEM-F12 | GIBCO | Cat# 12400-24 |
| Ampicillin/streptomycin | Biological industries | Cat# 03-031-1B |
| 2-mercaptoethanol | Sigma-Aldrich | Cat# M6250 |
| Triton X-100 | Sigma-Aldrich | Cat# 329830772 |
| BSA | Sigma-Aldrich | Cat# A9418 |
| PBS (10X) | Biological industries | Cat# 02-024-5A |
| Critical commercial assays | | |
| NF-κB (p65) transcription factor assay Kit | Cayman Chemical | Cat# 10007889 |
| Exo-spin™ buffer | Cell Guidance System | Cat# EX01 |
| Pierce™ BCA protein assay Kit | Thermo Fisher Scientific | Cat# 22237 |
| MILLIPLEX map rat cytokine magnetic bead (TNF, IL-6, IL-1β, and IFNγ) | Merck | Cat# RECYTMAG-65K |
| FITC annexin V apoptosis detection Kit | BD Pharmingen | Cat# 556547 |
| MTS assay | Promega | Cat# G109A |
|  |  |  |
| Deposited data | | |
|  |  |  |
| Experimental models: Cell lines | | |
| DITNC1 | ATCC | ATCC CRL-2005 |
| CAD cells ("CATH.a-differentiated") | ECACC General Collection | 08100805 |
|  |  |  |
| Experimental models: Organisms/strains | | |
| Male BALB/c mice | Instituto de Salud Pública Chile (ISP) | <https://www.ispch.cl/> |
| Wistar neonatal rats | Animal facility at Universidad  de Chile | CBA #1123 FMUCH |
|  |  |  |
| Oligonucleotides | | |
|  |  |  |
| Recombinant DNA | | |
|  |  |  |
| Software and algorithms | | |
| GraphPad Prism (version 9.0) | GraphPad Software | https://www.graphpad.com |
| Software Q-View | Quansys Biosciences | https://www.quansysbio.com/imaging/q-view-software/ |
| FlowJo version 10.8.0. | Becton, Dickinson & Company | https://www.flowjo.com/ |
| ImageJ | National Institutes of Health | https://imagej.nih.gov/ij/ |
|  |  |  |
